# Guard cell‐specific glycine decarboxylase manipulation affects Arabidopsis photosynthesis, growth and stomatal behavior

**DOI:** 10.1111/nph.70124

**Published:** 2025-04-11

**Authors:** Hu Sun, Nils Schmidt, Tracy Lawson, Martin Hagemann, Stefan Timm

**Affiliations:** ^1^ Plant Physiology Department University of Rostock Albert‐Einstein‐Straße 3 D‐18059 Rostock Germany; ^2^ University of Essex Wivenhoe Park Colchester CO4 3SQ UK

**Keywords:** Arabidopsis, environmental acclimation, glycine decarboxylase, guard cells, photorespiration, photosynthesis, stomata

## Abstract

- Photorespiration is a mandatory metabolic repair shunt of carbon fixation by the Calvin–Benson cycle in oxygenic phototrophs. Its extent depends mainly on the CO_2_ : O_2_ ratio in chloroplasts, which is regulated via stomatal movements. Despite a comprehensive understanding of the role of photorespiration in mesophyll cells, its role in guard cells (GC) is unknown. Therefore, a key enzyme of photorespiration, glycine decarboxylase (GDC), was specifically manipulated by varying glycine decarboxylase H‐protein (GDC‐H) expression in Arabidopsis GC.
- Multiple approaches were used to analyze the transgenic lines growth, their gas exchange and Chl fluorescence, alongside metabolomics and microscopic approaches.
- We observed a positive correlation of GC *GDC‐H* expression with growth, photosynthesis and carbohydrate biosynthesis, suggesting photorespiration is involved in stomatal regulation. Gas exchange measurements support this view, as optimized GC photorespiration improved plant acclimation toward conditions requiring a high photorespiratory capacity. Microscopic analysis revealed that altered photorespiratory flux also affected GC starch accumulation patterns, eventually serving as an underlying mechanism for altered stomatal behavior.
- Collectively, our data suggest photorespiration is involved in the regulatory circuit that coordinates stomatal movements with CO_2_ availability. Thus, the manipulation of photorespiration in GC has the potential to engineer crops maintaining growth and photosynthesis under future climates.

## Introduction

To enable the biosynthesis of organic compounds, CO_2_ must enter the intracellular air space of leaves and ultimately reach the chloroplasts and site of carbon fixation. This gas exchange is facilitated by stomata, which evolved more than 400 million years ago (Edwards *et al*., 1992, 1998). Stomata are microscopic, adjustable pores on the leaf surface that regulate CO_2_ influx, while, at the same time, controlling water loss through transpiration (Vavasseur & Raghavendra, 2005; Lawson, 2009; Santelia & Lawson, 2016). Thus, appropriate regulation of stomatal movement is crucial for maintaining plant productivity and water use efficiency (WUE), especially in response to fluctuations in temperature, light intensity, water availability and CO_2_ concentration (Pankasem *et al*., 2024). Stomatal aperture is controlled by two guard cells (GCs) surrounding the stomatal pore, which change in turgor and consequently volume to adjust aperture size (Araújo *et al*., 2011; Lawson & Blatt, 2014). Over the past centuries, significant progress has been made in understanding the development, structure and physiology of stomata (Daloso *et al*., 2016; Santelia & Lawson, 2016). Many external (i.e. atmospheric pressure, light availability and quality, temperature, water availability and humidity, and CO_2_) and internal (i.e. abscisic acid, circadian rhythms, GC ion transport, plant hormones and sugar concentrations) factors affect stomatal behavior (Inoue & Kinoshita, 2017; Jezek & Blatt, 2017; Lawson & Matthews, 2020). However, three main environmental factors regulate stomatal movement during the day. First, illumination typically induces stomatal opening to support photosynthesis. Second, water availability determines the extent to which stomata remain open. Third, CO_2_, often in combination with light, influences stomatal movement over the longer term, with internal CO_2_, *C*_i_, thought to regulate stomatal behavior with mesophyll photosynthesis (Mott, 1988). The mechanisms by which GC sense changes in CO_2_ and translate these signals into adjustments in metabolism are a matter of intense research (Engineer *et al*., 2016; Takahashi *et al*., 2022). Although several factors of the signaling cascade have been identified (will be discussed later), the actual CO_2_ sensor is still unknown. Additionally, the coordination between GC and mesophyll cell (MC) metabolism and CO_2_ demands is still not fully understood (Santelia & Lawson, 2016).

Stomata open upon illumination in most plants to facilitate CO_2_ uptake for mesophyll photosynthesis. Blue light and red light drive this process, with blue light having a dominant, photosynthesis‐independent effect at low intensities (*c*. 10 μmol m^−2^ s^−1^). Red light acts at higher intensities, aligning with photosynthesis demands, and is thought to be key in linking MC CO_2_ needs with stomatal behavior, that is stomatal conductance (*g*_s_). Interestingly, the coordination between photosynthesis and *g*_s_ was recently shown to be regulated through *C*_i_, by both *C*_i_‐dependent and *C*_i_‐independent mechanisms (Taylor *et al*., 2011). Chloroplasts regulate red light responses, but it remains unclear whether this applies specifically to MC or GC chloroplasts (Lawson, 2009). Guard cell photosynthesis, although debated, produces ATP and NADPH via the chloroplast electron transport chain (Lawson *et al*., 2002, 2003; Lawson, 2009) and supports blue light‐induced opening (Assmann *et al*., 1985; Santelia & Lawson, 2016). Guard cell chloroplasts also host a functional Calvin–Benson (CB) cycle, yet the extent of GC photosynthetic contribution compared with MC remains uncertain (Lawson, 2009; Lawson *et al*., 2014). Stomatal closure during water shortage is mediated by abscisic acid (ABA), synthesized within GC using endogenous enzymes. Abscisic acid synthesis can be self‐stimulated (Munemasa *et al*., 2015). Light‐induced opening and ABA‐mediated closure rely on transporter activities for ion movements regulated via distinct signal perception mechanisms: phototropins for blue light and phytochromes or photosynthetic pigments for red light, while the ABA signal is translated via the ABA‐signaling pathway (Chen *et al*., 2012). Stomatal opening occurs through GC turgor is increasingly driven by the osmotically active substance accumulation, mainly K^+^, Cl^−^, malate and sucrose. The proton motive force, generated by H^+^‐ATPase activity, powers ion transport, while sucrose metabolism supplies cytosolic energy and redox equivalents, fueling other GC processes (Roelfsmea & Hedrich, 2005; Gaxiola *et al*., 2007; Daloso *et al*., 2016). The metabolism of organic acids (malate, fumarate and pyruvate) and carbohydrates (starch and sucrose) in GC is also vital for stomatal dynamics (Araújo *et al*., 2011; Chen *et al*., 2012; Li *et al*., 2014).

In recent years, the significance of GC starch accumulation and its turnover has become a focus of research (Dang *et al*., 2024). Early observation showed the presence of starch granules correlates with stomatal aperture (Lloyd, 1908), suggesting starch is key to maintaining GC movements. Usually, starch is synthesized in plastids of all cell types, including photosynthetically active and heterotrophic, nonphotosynthesizing tissues. Guard cell starch metabolism differs from MC, with starch granules being rapidly degraded upon illumination to produce osmotic compounds supporting opening. Recent findings suggest that hydrogen peroxide (H_2_O_2_) is involved in the remobilization of GC starch under optimal conditions, too (da Silva *et al*., 2024; Shi *et al*., 2024). Unlike MC, GC retains starch in darkness, crucial for early opening stages. Structural differences between GC and MC chloroplasts (e.g. reduced thylakoid structures) indicate distinct roles (Vavasseur & Raghavendra, 2005; Lawson, 2009; Flütsch *et al*., 2020). However, experimental evidence highlights the importance of GC photosynthesis in stomatal function, involving CO_2_ fixation via the CB cycle and phosphoenolpyruvate carboxylase (PEPC), although the carbon contribution from each pathway remains unclear (Shimazaki & Zeiger, 1985; Shimazaki *et al*., 1989; Lawson, 2009; Lawson *et al*., 2014; Daloso *et al*., 2015).

In addition to light intensity and water availability, stomata also respond to external CO_2_ concentrations. Experimentally elevated CO_2_ in the atmosphere reduced stomatal aperture, while lower CO_2_ opens stomata (Negi *et al*., 2008; Engineer *et al*., 2016). Guard cell sensing of CO_2_ and aligning it with MC photosynthesis demand was thought to be based on *C*_i_; however, recent evidence suggests a requirement and intense interplay between *C*_i_‐dependent and *C*_i_‐independent mechanisms (Taylor *et al*., 2024). Studies indicate that isolated GC can respond to CO_2_ changes, suggesting that they possess the necessary components for CO_2_ perception and signaling (Weyers *et al*., 1983; Edwards & Bowling, 1985; Hu *et al*., 2010). The current signaling cascade involves carbonic anhydrases (CA1 and CA4), which mediate high CO_2_‐induced stomatal closure through bicarbonate (HCO_3_^−^) as a messenger (Hu *et al*., 2010, 2015). Other regulatory components include protein kinases (HT1 – high leaf temperature 1, OST1 – open stoma 1), anion channels (SLAC1 – slow anion channel‐associated 1, QUAC1 – quick anion channel 1) and transport proteins (RHC1 MATE – resistant to high carbon dioxide 1 multidrug and toxin extrusion transporter), which affect different stages of the signaling process (Engineer *et al*., 2016). CO_2_ signaling also intersects with ABA pathways, as suggested by studies on ABA receptor mutants (PYR/RCARs – pyrabactin resistance/regulatory component of ABA receptors) and PP2C protein phosphatases (*abi1‐1* and *abi2‐1*). However, the nature of this interaction remains enigmatic (Engineer *et al*., 2016). Ca^2+^ ions may also act as downstream messengers in this regulatory network, supported by the identification of the ABA‐insensitive mutant *gca2* (growth controlled by ABA; Young *et al*., 2006), although the role of *GCA2* requires further investigation.

In plants, the CB cycle and photorespiration are tightly regulated by the CO_2_ : O_2_ ratio (Busch, 2020; Fernie & Bauwe, 2020) in the chloroplasts. Since GCs contain RuBisCO and are capable of performing photosynthesis (Reckmann *et al*., 1990; Cardon & Berry, 1992; Lemonnier & Lawson, 2024), functional photorespiration is essential to metabolize 2‐phosphoglycolate (2PG), the major byproduct of RuBisCO's oxygenase activity, too. Photorespiration recycles carbon and phosphorus locked in 2PG and prevents its inhibitory effects on key enzymes of carbon utilization such as triosephosphate isomerase, SBPase and phosphofructokinase (Kelly & Latzko, 1976; Flügel *et al*., 2017; Li *et al*., 2019). Additionally, photorespiration detoxifies intermediates such as glycolate, glyoxylate and glycine, which otherwise impair RuBisCO activation, PSII redox balance and manganese homeostasis (Timm & Hagemann, 2020). Beyond the removal of critical intermediates, especially 2PG, photorespiration is vital for several other cellular processes. This includes the biosynthesis of certain amino acids to support other types of metabolism, maintaining subcellular redox balances and a major impact on nitrogen and sulfur metabolism (Foyer *et al*., 2009; Bloom *et al*., 2010; Abadie & Tcherkez, 2019; Busch, 2020; Fu *et al*., 2023). Photorespiration also releases H_2_O_2_ in the peroxisomes, a signaling molecule recently suggested to be involved in the regulation of stomatal aperture (da Silva *et al*., 2024; Shi *et al*., 2024). Furthermore, the photorespiratory glycine‐to‐serine conversion catalyzed by glycine decarboxylase (GDC) in conjunction with serine hydroxymethyltransferase 1 is connected to one‐carbon metabolism, recently suggested to form an overlapping metabolic network with nitrogen and sulfur metabolism (Rosa‐Téllez *et al*., 2024). The capacity of photorespiration is considered significant in maintaining plant metabolism under environmental fluctuations that affect intracellular CO_2_ : O_2_ ratios (Timm *et al*., 2019; Meacham‐Hensold *et al*., 2024; Sun *et al*., 2024). To date, the role of photorespiration in GC has remained unexplored, while investigating this could reveal how GC adapt their metabolism to fluctuating CO_2_ : O_2_ ratios and, possibly, align these changes with MC demands. We hypothesize that GC photorespiration eventually contributes to the *C*_i_‐dependent regulation mechanism of stomatal movements and communication with the mesophyll. Therefore, we tested whether, and how, manipulation of photorespiration's central enzyme glycine decarboxylase H‐protein (GDC‐H) expression in GC has any significant effect on leaf physiology, metabolism and, particularly, GC movements. Understanding and rationally modifying the underlying regulatory circuit could aid in engineering crop plants with higher growth rates and yields under future climates.

## Materials and Methods

### Plant material, growth conditions and growth parameters

During this study, *Arabidopsis thaliana* L. (Arabidopsis) ecotype Columbia 0 (Col‐0) served as the wild‐type (WT) reference and background to produce GC‐specific transgenic lines with modulated GDC‐H expression. All seeds were surface‐sterilized with chloric acid, sown on a soil (Type MiniTray; Einheitserdewerk, Uetersen, Germany) and vermiculite mixture (4 : 1) and incubated for 2 d at 4°C to break dormancy. Unless stated otherwise, plant growth was performed in controlled environment chambers (Percival) under the following standard conditions: photoperiod 12 h : 12 h, 22°C : 20°C, day : night, *c*. 120 mmol m^−2^ s^−1^ irradiance, 400 ppm CO_2_ and *c*. 70% relative humidity. When specified, the CO_2_ concentration was increased to 3000 ppm or the photoperiod changed (10 : 14 or 14 : 10 h day : night), with otherwise equal parameters. During cultivation, plants were watered with 0.2% Wuxal liquid fertilizer (Aglukon) weekly. For all physiological experiments, plants at growth stage 5.1 (Boyes *et al*., 2001) were used. The following quantitative growth parameters were determined from six to 10 biological replicates per genotype: (1) rosette diameter, longest possible distance of the fully expanded rosette; (2) total leaf count, only considering fully expanded rosette leaves; and (3) fresh and dry (dried for *c*. 24 h at 100°C) weights of the entire plant rosette.

### Cloning and plant transformation procedures

In order to obtain transgenic lines with GC‐specific GDC manipulations, we first PCR‐amplified a 1141‐bp genomic fragment of the GC preferential GC1 promoter (Yang *et al*., 2008) from WT DNA using oligonucleotides (sequences in Supporting Information Table S5) *At*GC1_S1141_SacI (P950) and *At*GC1‐AS‐BamHI (P951) and the entire GDC‐H coding sequence (489 bp; Kopriva & Bauwe, 1995) from *Flaveria pringlei* cDNA using oligonucleotides *Fp*GDCH‐S‐PstI (P965) and *Fp*GDCH‐AS‐PstI (P966). The resulting fragments (*At*GC1‐1141 and *Fp*GDCH) were ligated into the vector pJET2.1 (Thermo Fisher Scientific, Schwerte, Nordrhein‐Westfalen, Germany) and sequenced for verification. Next, the *At*GC1‐1141 SacI‐BamHI promoter fragment was excised from pJET2.1:*At*GC1 and ligated into the binary plant transformation vector pGREEN0229, containing the 35S terminator, previously introduced through restriction and ligation from the 35S cassette (EcoRI and EcoRV), to obtain pG0229:*At*GC1:35STer. Finally, the *Fp*GDCH PstI:PstI fragment was excised from pJET2.1:*Fp*GDCH and ligated into pG0229:*At*GC1:35STer in sense (pG0229:AtGC1:*Fp*GDCH:35STer‐sense) and antisense (pG0229:*At*GC1:*Fp*GDCH:35STer‐antisense) orientation to obtain the GC‐specific overexpression and antisense suppression constructs (Fig. S1A,B). Both constructs were introduced into *Agrobacterium tumefaciens* strain GV3101 + pSOUP and used for plant transformation (Clough & Bent, 1998). The resulting phosphinotricine (Basta)‐resistant plants were PCR‐verified and stable T3 lines from at least three independent transformation events (designated as GC1:*Fp*GDCH sense lines SL1, SL4 and SL7 and GC1:*Fp*GDCH antiline AL4, AL5 and AL9, respectively) propagated and used for comprehensive characterization.

### Verification of transgenic lines, RT‐PCR and immunological studies

To verify the genomic integration of the constructs, leaf DNA, isolated following standard procedures, was PCR‐amplified (1 min at 94°C, 1 min at 58°C and 1.0 min at 72°C; 35 cycles) with primers specific to the *Fp*GDCH fragment (P965 for sense and P966 for antisense orientation) in combination with the 35S terminator (P807). The *S16* gene was amplified (1 min at 94°C, 1 min at 58°C and 30 s at 72°C; 35 cycles) using oligonucleotides *S16*‐forward (P444) and *S16*‐reverse (P445) as control (Fig. S1C). The functionality of the integrated overexpression and antisense constructs was first verified on the whole leaf basis via semiquantitative reverse transcription polymerase chain reaction, using 2.5 μg leaf RNA for cDNA synthesis (Nucleospin RNA plant kit (Macherey‐Nagel) and RevertAid cDNA synthesis kit (MBI Fermentas)). The oligonucleotide combination *Fp*GDCH‐S‐PstI (P965) and *Fp*GDCH‐AS‐PstI (P966) was used to amplify the full‐length *Fp*GDCH transcript (489‐bp PCR product). The constitutively expressed 40S ribosomal protein *S16* gene was amplified with oligonucleotides *S16*‐forward (P444) and *S16*‐reverse (P445) as a positive control. Second, changes in GDC‐H expression were further analyzed by immunoblotting. Briefly, protein extracts of MC and GC preparations, in comparison with whole leaf preparations, were separated by SDS‐PAGE and gel blotting experiments performed according to standard protocols. Changes in the protein abundances were detected using specific antibodies for GDC‐H (Kopriva *et al*., 1996), using PGLP1 (Flügel *et al*., 2017) as a calibration control. ImageJ was used to determine the band intensities as a measure of altered protein abundances from at least three independent immunoblots.

### Isolation of mesophyll and guard cell protein extracts

To obtain MC‐ and GC‐specific protein extracts, we enriched both cell fractions following the protocol described by Lawrence *et al*. (2018). Briefly, we used 5‐wk‐old plants grown under standard conditions. Scotch tape was attached to fully expanded leaves on either the abaxial (lower, for GC enrichment) or adaxial (upper, for mesophyll enrichment) side of the leaves. Subsequently, the peels (*c*. 30 per genotype) were gently removed and immediately frozen in liquid nitrogen. For protein extraction, we followed the protocol described earlier (Lawrence *et al*., 2018). Subsequent to the dissolution of the proteins in buffer, their concentration was determined using the BCA Protein Assay Kit (Thermo Scientific) according to the manufacturer's instructions. For SDS‐PAGE and immunoblotting analysis, we used 5 μg of the respective protein extracts.

### Standard gas exchange measurements

Photosynthetic gas exchange parameters were determined on a Li‐6400xt Portable Photosynthesis System (Li‐Cor, Lincoln, NE, USA), using plants grown under standard conditions at a light intensity of *c*. 120 μmol m^−2^ s^−1^. The following settings were used as the standard: 1000 μmol m^−2^ s^−1^ photon flux density (10% blue light), 25°C block temperature, 400 ppm CO_2_, 300 μmol s^−1^ flow rate and 50–70% relative humidity. To determine CO_2_ compensation points at various O_2_ concentrations (3%, 21% and 40%; balanced with N_2_), *A*/*C*_i_ curves were measured (400, 300, 200, 100, 50, 25, 0, 400 μl l^−1^ CO_2_). Mean values ±SD of net CO_2_ uptake rates (*A*_N_), CO_2_ compensation points (Γ), stomatal conductance (*g*_s_), intercellular CO_2_ concentrations (*C*_i_), transpiration rates (*E*) and intrinsic WUE (WUE_int_) were consistently calculated from the final 400 μl l^−1^ CO_2_ step from at least six biological replicates. Oxygen inhibition of *A*_N_ was calculated from measurements at 21% and 40% O_2_ using the following equation: O_2_ inhibition = (*A*_21_–*A*_40_)/*A*_21_ × 100. Calculation of *γ* (a measure of the photorespiratory CO_2_‐release) was performed by linear regression of the *Γ*‐vs‐oxygen concentration curves and is given as slopes of the respective functions. Light–response curves were measured using ambient air CO_2_ and O_2_ levels, using varying light intensities (1759, 1144, 757, 488, 236, 143, 62, 36, 0 μmol m^−2^ s^−1^).

### PSI and PSII Chl fluorescence measurements

Chlorophyll fluorescence measurements were performed on a Dual‐PAM 100 (Heinz Walz, Effeltrich, Germany) to determine selected PSI and PSII parameters. We measured Chl fluorescence from the adaxial side of the leaf, and it should be noted that PSI refers to the P700 measurement on the whole leaf tissue, and PSII is a fluorescence measurement at a certain depth in the leaf tissue. Initial *F*_v_/*F*_m_ (maximum quantum efficiency of PSII) and *P*_m_ (maximum photo‐oxidizable P700) values were recorded following a 10‐min dark adaptation period. Plants were then exposed to 1000 μmol photons m^−2^ s^−1^ for 10 min to fully induce photosynthesis. Subsequently, light–response curves were measured at varying light intensities (1759, 1144, 757, 488, 236, 143, 62, 36, 0 μmol photons m^−2^ s^−1^) at 400 ppm CO_2_ and 21% O_2_.

### Determination of metabolite levels via LC‐MS/MS, GC and spectrophotometric measurements

Abundances of primary metabolites were quantified by liquid chromatography coupled to tandem mass spectrometry (LC‐MS/MS) and gas chromatography analysis, using leaf tissue from plants at the end of the day (11 h of illumination). Whole plant rosettes were harvested under illumination within the growth cabinets, immediately frozen in liquid nitrogen and stored at −80°C. Before analysis, plants were freeze‐dried by lyophilization and aliquoted (*c*. 3–5 mg dry weight). Extraction and LC‐MS/MS measurements were carried out using LC‐MS grade chemicals as described in Arrivault *et al*. (2009, 2015) and the modifications specified in Reinholdt *et al*. (2019). The total amino acid content is a sum parameter of the following representatives: alanine, arginine, asparagine, cysteine, cystine, glutamate, glutamine, glycine, histidine, isoleucine, leucine, lysine, methionine, phenylalanine, proline, serine, threonine, tryptophan, tyrosine and valine. The total organic acid content is a sum parameter of the following representatives: aconitate, citrate, fumarate, GABA, isocitrate, lactate, malate and succinate.

Soluble sugars were extracted from homogenized plant material in 800 μl of 80% ethanol, containing 20 μg of ribitol as internal standard, at 80°C for 30 min. After centrifugation (20 000 ***g***, 10 min, 4°C), the supernatant was dried by lyophilization. The pellet was used for starch quantification through spectrophotometric analysis using enzymatic assays in ethanolic extracts described elsewhere (Cross *et al*., 2006). Dried pellets containing soluble carbohydrates were resuspended in 65 μl of pyridine containing 20 mg ml^−1^ methoxylamine at 30°C for 90 min. Subsequently, 35 μl of N‐methyl‐N‐trimethylsilyl‐trifluoracetamide was added, and the samples incubated at 65°C for 90 min and briefly centrifuged (20 000 ***g***, 1 min). After cooling, aliquots of the supernatants were analyzed on the gas chromatograph 6890 N GC System (Agilent Technologies, Waldbronn, Baden‐Württemberg, Germany), equipped with a TG‐5MS column (Thermo Scientific). Samples were injected with an inlet temperature of 250°C and 40 ml min^−1^ nitrogen gas flow at a split ratio of 20 : 1. A flow of 1.8 ml min^−1^ nitrogen gas (mobile phase) and an average velocity of 45 cm s^−1^ at 172 kPa were used. The oven temperature was initially set to 160°C for 2 min, following a gradual increase to 190°C (10°C min^−1^) for 5 min. Subsequently, the temperature was increased to 200°C in a step gradient (1°C min^−1^) within 15 min, following a gradual increase to 280°C (15°C min^−1^) for 5 min. The Flame Ionization Detector was set to 250°C, 40 ml min^−1^ hydrogen and 400 ml min^−1^ compressed air, and a makeup gas flow (nitrogen) of 8.2 ml min^−1^. Authentic standards (GC grade) were used for qualitative and quantitative analyses.

### Guard cell properties and guard cell starch content

To determine selected GC parameters and starch contents, we used epidermal peels from plants grown under standard conditions in the middle of the photoperiod (6‐h illumination). Briefly, nail polish was applied on the abaxial side of the leaves and incubated for 10 min. Furthermore, the epidermis was separated from leaves (six replicates per line), put on a slide with water and covered with a coverslip. The prepared slides were subjected to microscopic analysis on a U‐LH100HG microscope (Olympus Corp., Tokyo, Japan) to determine selected GC properties using the manufacturer's software. The GC starch content was quantified in epidermal peels (four biological replicates per genotype, 10 GC per stained peel) as described in Flütsch *et al*. (2018) following propidium iodide staining. Images were acquired using the Keyence BZ‐X800 fluorescence microscope (Keyence Deutschland GmbH, Neu‐Isenburg, Germany) equipped with a Plan Fluorite 20‐100X LD PH objective (×100 magnification). Fluorescence was visualized using a BZ‐X filter GFP cube (exposure time 1/70 s). Images were captured with the BZ‐X800 Viewer software.

### Statistical analysis

Statistical differences were determined by ANOVA (SPSS Statistics 27, IBM). We used the term significant here if the change in question had been confirmed to be significant at the level of *, *P* < 0.05. The Metaboanalyst 6.0 platform (Ewald *et al*., 2024) was used to analyze and display the metabolic data.

## Results

### Isolation of guard cell‐specific Arabidopsis photorespiration mutants

**Fig. 1:**
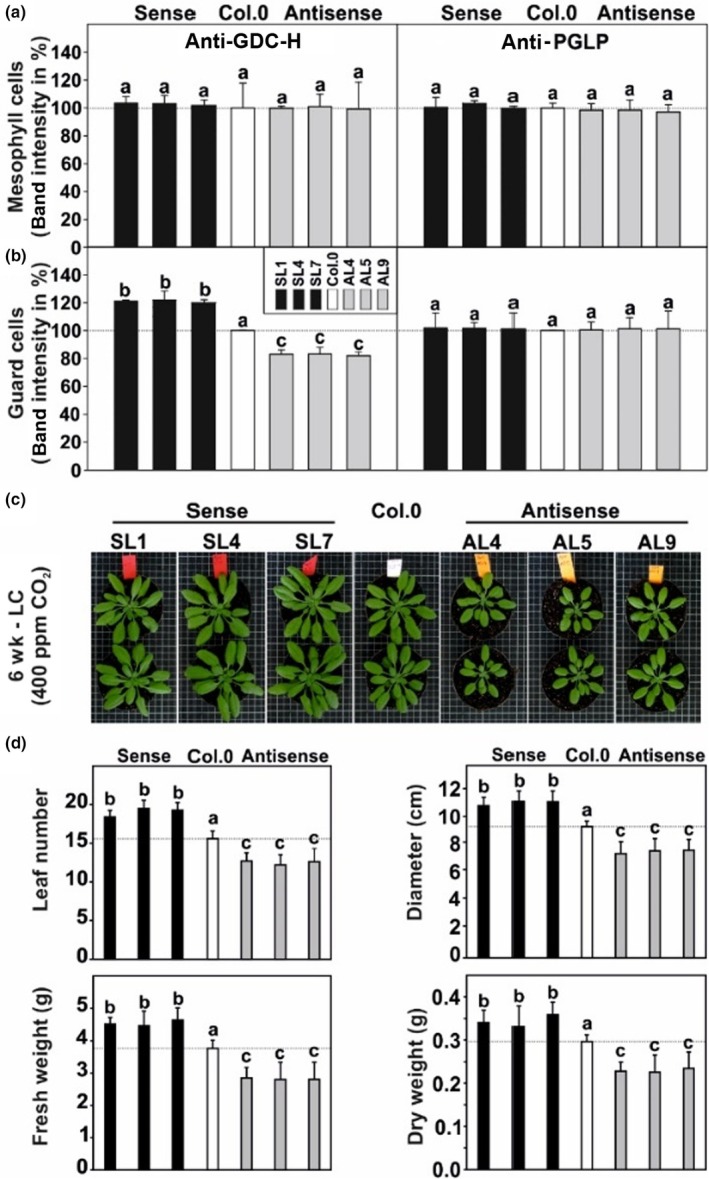
Glycine decarboxylase H‐protein (GDC‐H) expression, phenotype and growth of the transgenic, guard cell (GC)‐specific *GDC‐H* modified lines and the wild‐type (WT). Given are GDC‐H (left panels) and PGLP1 (right panels) protein abundances in (a) mesophyll cell (MC) and (b) GC preparations obtained from Arabidopsis plants grown in normal air to stage 5.1 (Boyes *et al*., 2001). Protein expression was quantified by densitometry and expressed in % band intensity (mean ± SD, from three individual immunoblots) of signals from the transgenic lines and the WT, which was arbitrarily set to 100% (immunoblot examples are given in Supporting Information Fig. S1). (c) Representative images of plants grown in normal air (LC – low carbon; 400 ppm CO_2_) in a 12 h : 12 h, day : night cycle (for pictures of plants grown in high CO_2_ and other photoperiods, see Fig. S2). (d) Selected growth parameters of all genotypes at the same age. Fresh weight/dry weight ratios: SL1 = 13.3 ± 0.9^a^; SL4 = 13.5 ± 0.8^a^; SL7 = 13.0 ± 0.7^a^; Col.0 = 12.7 ± 0.9^a^; AL4 = 12.5 ± 0.4^a^; AL5 = 12.4 ± 0.5^a^; and AL9 = 11.9 ± 0.8^a^. Given are means ± SD of at least eight biological replicates. Values that do not share the same letter are significantly different from each other as determined by ANOVA.

To investigate whether photorespiration is active in GC and involved in stomatal movements, we produced transgenic Arabidopsis lines with modified expression of GDC‐H. GDC‐H was chosen based on previous research demonstrating GDC is key in controlling carbon flux through photorespiration, influencing photosynthesis and growth (Timm *et al*., 2012a; Simkin *et al*., 2017; López‐Calcagno *et al*., 2019). To increase (overexpression) and decrease (antisense suppression) *GDC‐H* expression in GC, the full‐length sequence encoding the *F. pringlei* GDC‐H was cloned into the plant transformation vector pGREEN0229 (http://www.pgreen.ac.uk/). This was carried out under the control of the GC‐specific *GC1* promoter (Yang *et al*., 2008; Wang *et al*., 2014) and the CaMV 35S terminator, in both sense and antisense orientations (Fig. S1A,B). These constructs were transformed into Arabidopsis WT plants. Phosphinotricine (BASTA)‐resistant plants were PCR‐verified, propagated to stable T3 generations and used for characterization (Fig. S1C). Expression of the transgene was confirmed at the mRNA level (Fig. S1D), followed by tissue‐specific analysis of GDC‐H abundance through immunoblotting. As shown in Figs 1(a) and S1E, GDC‐H remained virtually unchanged in MC. However, in GC preparations, we observed increased (*c*. 20–22%) or decreased (*c*. 16–18%) GDC‐H abundance in the three overexpression lines (SL2, SL4 and SL7) and the three antisense lines (AL4, AL5 and AL9), respectively (Figs 1b, S1E). Notably, GC‐specific alterations did not affect the whole leaf GDC‐H abundance (Fig. S1E), confirming the specificity of the approach.

### Guard cell‐specific manipulation of photorespiratory GDC correlates with plant growth

Next, we investigated whether modified *GDC‐H* expression in GC affected plant growth. To this end, plants were cultivated with sufficient water supply under standard conditions (12 h : 12 h, day : night cycle) and analyzed. As shown in Fig. 1(c), we observed a correlation between GC‐specific GDC‐H abundance and growth. Visual comparisons indicated that the overexpression of *GDC‐H* stimulated plant growth, while reduced *GDC‐H* expression diminished it (Fig. 1c). It is important to note that growth changes were negligible under elevated CO_2_ conditions (3000 ppm), suppressing photorespiration (Fig. S2A, lower panel). Absolute biomass quantification under standard conditions revealed that total leaf numbers and rosette diameters were significantly increased in the overexpression lines and decreased in the antisense lines. These changes in leaf parameters translated to alterations in overall fresh weight (FW) and dry weight (DW), although the FW : DW ratios remained consistent across all genotypes (Fig. 1d). Interestingly, growth effects were consistent across different photoperiods (10 h : 14 h, day : night, 12 h : 12 h, day : night and 14 h : 10 h, day : night cycles, all at 400 ppm CO_2_); however, they appeared somewhat stronger in longer day–night cycles (Fig. S2B,C).

### Photosynthetic light reactions are unaffected by guard cell‐modulated photorespiration

Alongside the phenotyping experiments, we characterized the photosynthetic capacity of transgenic lines and WT plants. Furthermore, we used a combination of Chl fluorescence and gas exchange measurements to distinguish light reaction‐driven effects from those resulting from altered carbon fixation reactions, eventually occurring in response to changes in stomatal behavior, that is stomatal conductance (*g*_s_). No systematic changes emerged during the comparison between a number of PSII and PSI parameters. The maximum quantum yield of PSII (*F*_v_/*F*_m_) and the maximum oxidation of P700 at the PSI reaction center (*P*_*m*_) were invariable among the studied genotypes under standard growth conditions (Fig. S3A). These trends were stable over a wide range of light intensities, as the quantum yields of PSII (Y[II]) and PI (Y[I]) did not significantly differ from each other. The same tendencies were also observed when the relative electron transport rates (rETRII and rETRI) were calculated (Fig. S3B,C). Furthermore, no significant differences were detected in the nonphotochemical quenching of PSII, the cyclic electron flow around PSI (CET) or the acceptor (Y[NA]) and donor (Y[ND]) side limitation of PSI (Fig. S3D,E).

### Increased guard cell GDC‐H expression improves light‐dependent CO_2_ assimilation

**Fig. 2:**
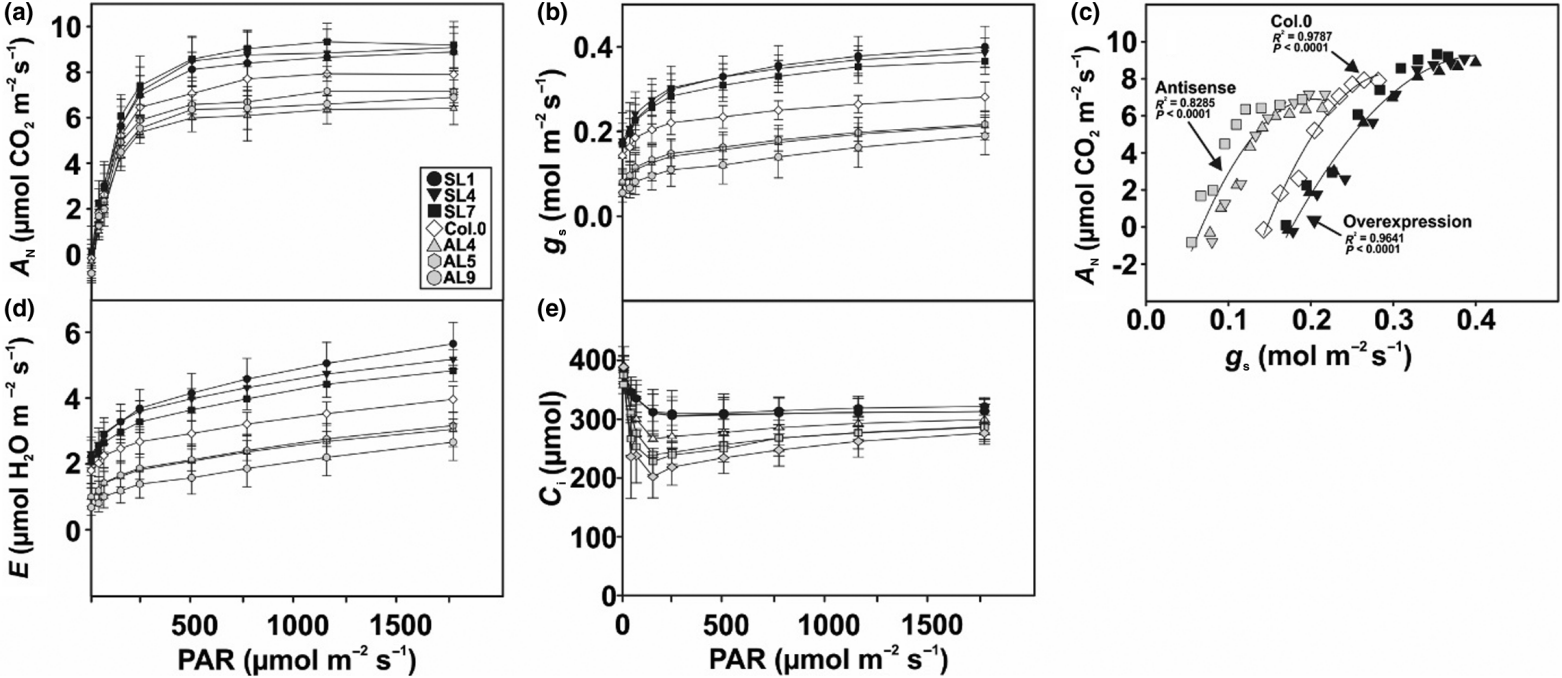
Light–response curves of the transgenic, guard cell‐specific glycine decarboxylase H‐protein modified lines and the wild‐type. Given are selected leaf gas exchange parameters as functions of varying light intensities determined of Arabidopsis plants at stage 5.1 (Boyes *et al*., 2001) grown under standard conditions. Given are the following: (a) net CO_2_ assimilation rate (*A*_N_); (b) stomatal conductance (*g*_s_); (c) correlation plot of *A*_N_ and *g*_s_; (d) transpiration rate (*E*); and (e) intracellular CO_2_ concentration [*C*_*i*_]. Shown are means ± SD of at least six biological replicates. Further parameters, all numerical values, and statistical evaluation are provided in Supporting Inormation Table S1.

Photosynthetic gas exchange parameters provided a different picture. First, we assessed the light acclimation capability of the transgenic lines and the WT through measurements of light–response curves. As summarized in Fig. 2 (numerical values in Table S1), we observed significantly increased net CO_2_ assimilation (*A*_N_), stomatal conductance (*g*_s_) and transpiration (*E*) in the overexpression plants, while all three measurements were lower in the antisense lines than in the WT. Interestingly, changes in *A*_N_ were strongly dependent on *g*_s_, as revealed by correlation analysis (Fig. 2a–d). The estimation of the maximum photosynthetic rate (*A*_max_) from the light–response curves was significantly greater in the overexpressors, while decreases were observed in the antisense lines compared with the WT. Similarly, initial slopes of the light–response curves (*α*_p_) were accelerated in overexpressors, but significantly unchanged in the suppressor lines compared with the WT controls (Table S2). Interestingly, higher and lower *g*_s_ in the transgenic plants also affected the intracellular CO_2_ concentrations (*C*_i_), which were increased and decreased in the overexpression and antisense lines, respectively (Fig. 2e). The WUE_int_ did not significantly vary among the studied genotypes (Table S1).

### Guard cell GDC‐H expression specifically impacts O_2_ ‐dependent leaf gas exchange

**Fig. 3:**
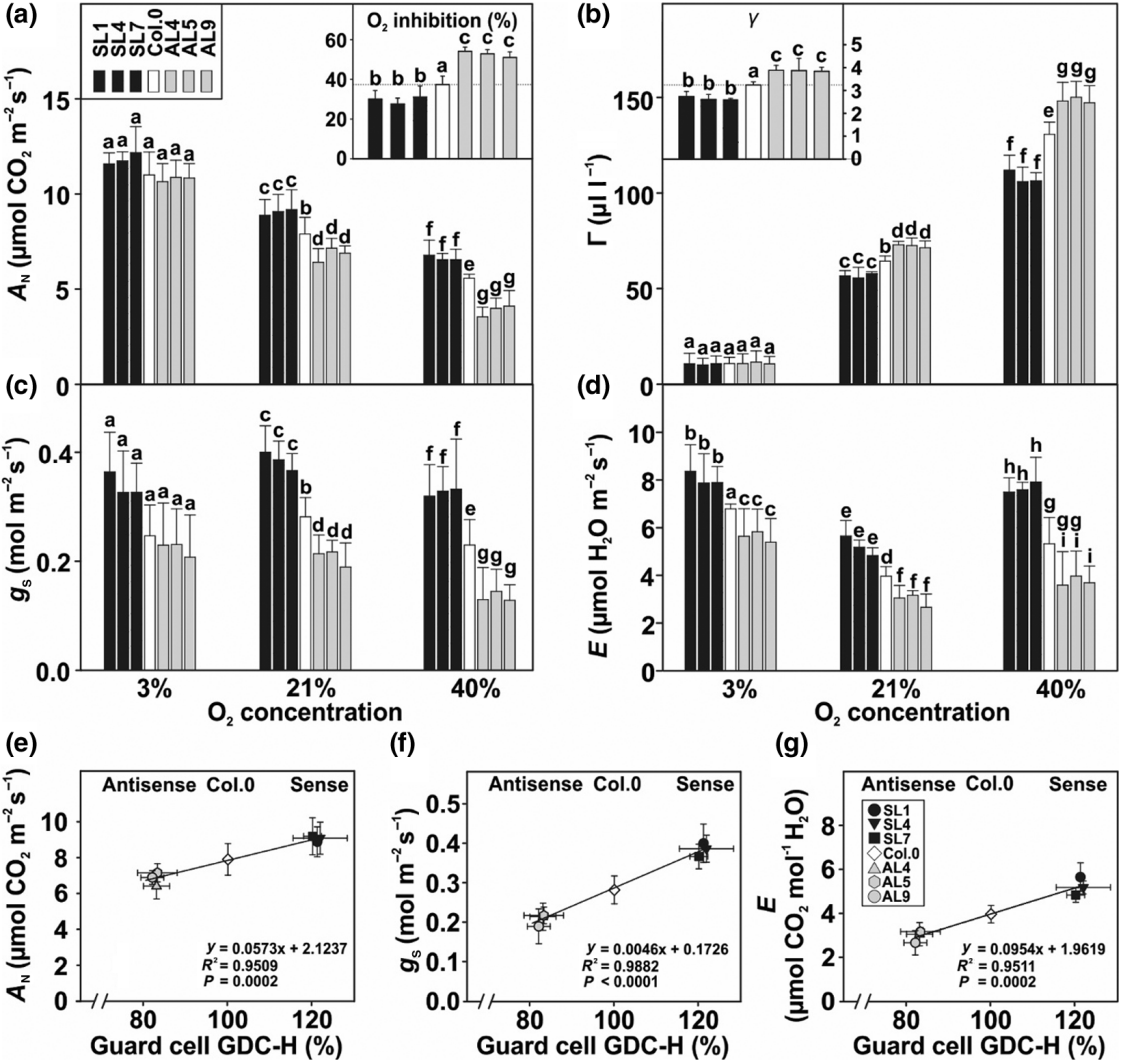
Oxygen‐dependent gas exchange parameters of guard cell (GC)‐specific glycine decarboxylase H‐protein (GDC‐H) modulated lines and the wild‐type (WT). Displayed are selected leaf gas exchange parameters at three different O_2_ concentrations (3%, 21% and 40%, balanced with N_2_) determined using all Arabidopsis genotypes grown under standard growth conditions to stage 5.1 (Boyes *et al*., 2001). Given are the following: (a) net CO_2_ assimilation rates (*A*_N_) and O_2_ inhibition of *A* (insert, the overexpression lines were less (−16.6 to −26.2%), while antisense lines were more (+36.6 to +34.8%) inhibited at increased O_2_); (B) CO_2_ compensation points (Γ) and *γ*, slopes of Γ – vs – O_2_ curves, (insert); (C) stomatal conductance (*g*_s_); and (d) transpiration rates (*E*). Shown are means ± SD of at least six biological replicates. Values that do not share the same letter are significantly different from each other as determined by ANOVA. Correlation analysis between GC GDC‐H protein expression and (e) *A*_N_, (f) *g*_s_ and (g) *E*, measured in 21% O_2_.

Given our transgenic approach directly targeted photorespiration, we next measured CO_2_–response curves at three different O_2_ concentrations (3%, 21% and 40%) to follow responses with various photorespiratory flux requirements. Net CO_2_ assimilation rates (*A*_N_) and CO_2_ compensation points (Γ) were significantly invariant among the genotypes at 3% O_2_, suppressing photorespiration (Fig. 3a,b). However, at 21% O_2_, *A*_N_ was elevated (up to *c*. 16.3%) or lowered (up to *c*. 18.9%) in the overexpression and antisense lines, respectively (Fig. 3a). In agreement, Γ was significantly decreased (up to *c*. 13.6%) in the overexpression lines and increased (up to *c*. 13.3%) in the antisense lines (Fig. 3b). These tendencies were pronounced at 40% O_2_, as the overexpressors displayed increased *A*_N_ (up to *c*. 22%) and a decrease in Γ (up to *c*. 19%), while *A*_N_ of the antisense lines was reduced (between 26.2% and 36.4%) alongside a significant rise in Γ (up to *c*. 15%) (Fig. 3b). The deduced values of O_2_‐inhibition of *A*_N_ largely supported the described tendencies, given the overexpression lines were less and the antisense lines more inhibited by high O_2_ compared with the WT (Fig. 3a). We also calculated *γ*, the slope of the Γ‐vs‐O_2_ concentration function, representing a measure of the photorespiratory CO_2_ release. This parameter was significantly lower in the overexpression lines and elevated in the antisense suppressors (Fig. 3b). Related to stomatal functioning, we assessed *g*_s_ and *E* in the same experiment. Similar to the above trends, *g*_s_ and *E* were increased in the overexpression lines and decreased in the antisense lines at all O_2_ concentrations (Fig. 3c,d). The only exception was statistically invariant *g*_s_ at 3% O_2_ suppressing photorespiration (Fig. 3c).

To verify whether the measured alterations in leaf gas exchange parameters correlate with the photorespiratory flux in GC, we plotted their GDC‐H amounts against selected photosynthetic parameters. Supporting our hypothesis, we found a strong positive correlation between GC GDC‐H expression and *A*_N_, *g*_s_ and *E* measured under the ambient air O_2_ concentration of 21%. Hence, all three parameters were lower in the antisense lines and higher in the overexpression mutants than in the WT (Fig. 3e–g).

### Optimized GC photorespiration increases the accumulation of leaf carbohydrates

To determine whether improved *g*_*s*_ and photosynthesis facilitate accumulation of photosynthates, eventually stimulating growth, we quantified total leaf abundances of selected carbohydrates. Three soluble sugars (sucrose, glucose and fructose) and transitory starch were measured in standard‐condition grown plants. As summarized in Table 1, soluble sugars were significantly elevated in the overexpression lines, but lower in the antisense lines than in the WT. Furthermore, transitory starch measured in the same material followed the described pattern, as it was increased in the overexpressors and reduced in the antisense lines (Table 1).

**Table 1:** Soluble sugars and starch in leaves of the transgenic lines and the wild‐type.

Genotype	Compound			
	Sucrose	Fructose	Glucose	Starch
SL1	**5.88 ± 0.89** ^**a**^	**2.88 ± 0.53** ^**b**^	**17.76 ± 3.16** ^**b**^	**152.32 ± 12.81** ^**b**^
SL4	**5.36 ± 0.52** ^**a**^	**2.73 ± 0.18** ^**b**^	**16.15 ± 1.19** ^**b**^	**159.53 ± 29.32** ^**b**^
SL7	**5.18 ± 0.29** ^**a**^	**2.68 ± 0.57** ^**b**^	**14.12 ± 1.53** ^**b**^	**145.62 ± 10.85** ^**b**^
Col.0	4.60 ± 0.28^b^	1.90 ± 0.26^a^	8.52 ± 2.35^a^	117.54 ± 18.48^a^
AL4	**3.89 ± 0.45** ^**c**^	**1.25 ± 0.24** ^**c**^	**4.37 ± 0.78** ^**c**^	**73.37 ± 18.63** ^**c**^
AL5	**3.95 ± 0.34** ^**c**^	**1.18 ± 0.20** ^**c**^	**3.48 ± 1.55** ^**c**^	**77.26 ± 15.16** ^**c**^
AL5	**3.60 ± 0.41** ^**c**^	**1.24 ± 0.21** ^**c**^	**3.99 ± 2.58** ^**c**^	**72.67 ± 12.42** ^**c**^

Arabidopsis plants were grown under environmentally controlled standard conditions in normal air (400 ppm CO_2_) to growth stage 5.1 (Boyes *et al*., 2001). Leaf material was harvested at the end of the day (EoD – 11‐h illumination) and used for quantification of selected soluble sugars by gas chromatography. Starch was measured spectrophotometrically, essentially using the same material. Values are means ± SD (*n* = 6) and are given in microgram per gram per dry weight. Values that do not share the same letter are significantly different from each other, as determined by ANOVA. Values in bold indicate *P* < 0.05.

### Primary metabolism in the GC‐specific transgenic lines responds with specific shifts associated with 3PGA and amino and organic acid metabolism

**Fig. 4:**
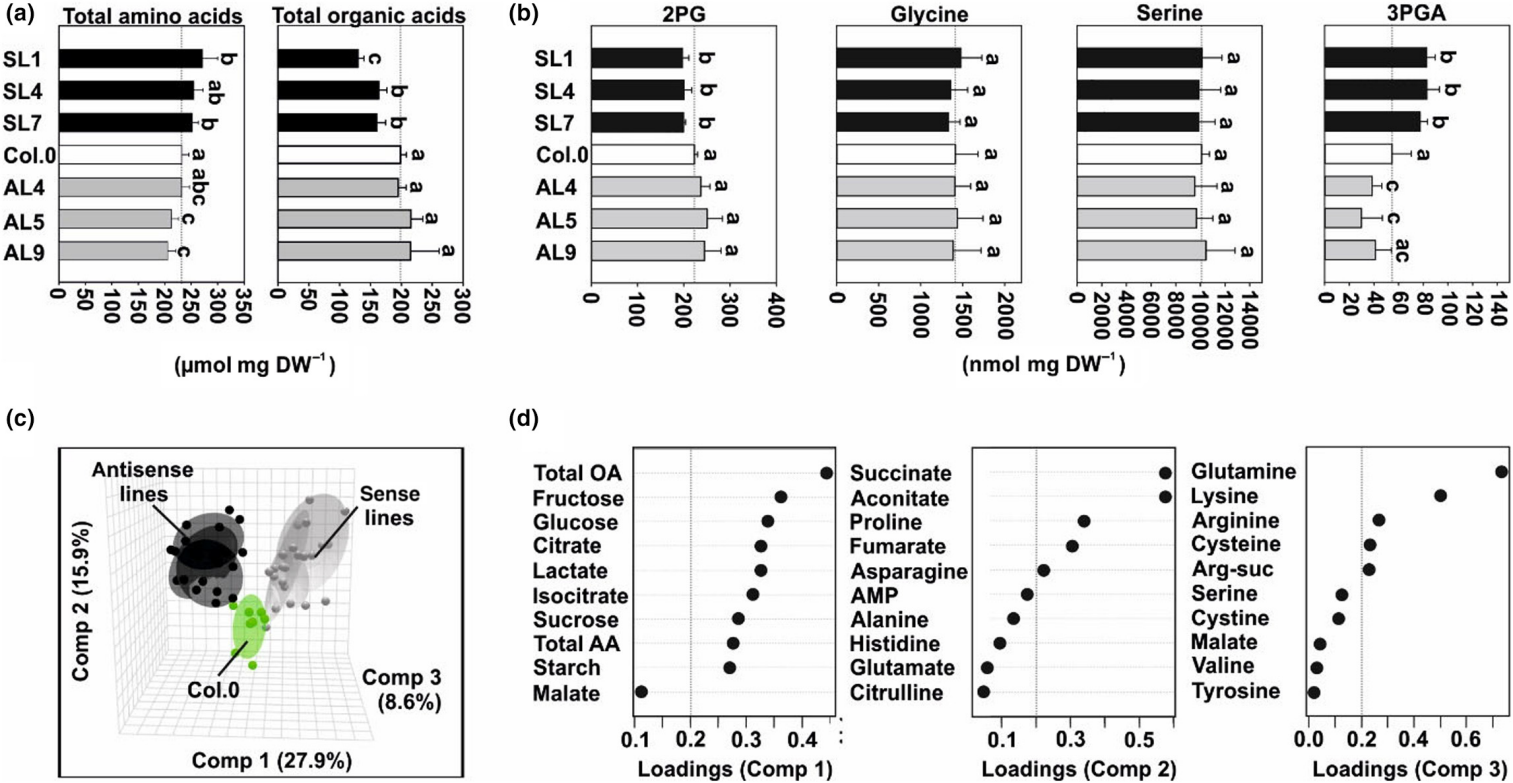
Overview of metabolite responses in guard cell (GC)‐specific glycine decarboxylase H‐protein (GDC‐H) modulated lines and the wild‐type (WT). Arabidopsis plants were grown under environmentally controlled standard conditions in normal air (400 ppm CO_2_) to growth stage 5.1 (Boyes *et al*., 2001). Leaf material was harvested at the end of the day (EoD – 11‐h illumination) and used for quantification of primary metabolites by LC‐MS/MS analysis. Given are (a) total soluble amino acid and organic acid contents, (b) selected intermediates of photorespiration, (c) principal component analysis (PCA) of all metabolite data, and (d) loadings of the first three components driving the cluster separation during PCA. Loadings exceeding the cutoff (±0.2, on the right of the dotted line) were considered to have a strong impact on cluster separation. Metabolite contents are means ± SD (*n* > 6) and are given in micromole (a) or nanomole (b) g^−1^ dry weight^−1^, respectively. Values that do not share the same letter are significantly different from each other as determined by ANOVA. AMP, adenosine monophosphate; Arg‐suc, l‐argininosuccinic acid; Comp, component; total AA, total amino acids; total OA, total organic acids.

Simultaneous to carbohydrate measurements, we quantified 35 further primary metabolites, including four photorespiratory intermediates (2PG, glycine, serine and 3PGA), through LC‐MS/MS. Only a few consistent, mutant‐specific alterations were seen in leaves at the end of the day from plants grown under standard conditions (Table S3). However, the overexpression lines generally tend to accumulate soluble amino acids (significant in SL1 and SL7), while organic acids were decreased. By contrast, antisense lines displayed somewhat opposite tendencies, with soluble amino acids being decreased (significant in AL5 and AL9) and organic acids were present in similar amounts as in the WT (Fig. 4a). Related to photorespiration, we found statistically invariant glycine and serine amounts among the genotypes and only slight decreases in 2PG in the overexpressors. Interestingly, 3PGA followed the same pattern described for carbohydrates (Table 1), as its contents were increased in overexpressors and decreased in antisense lines (except for AL9) (Fig. 4b).

To gain a general, comparative overview on the metabolic changes, we analyzed the full data set (GC, starch and LC‐MS/MS data) by principal component analysis (PCA). As shown in Fig. 4(c), metabolites in the different genotypes formed three different clusters, namely the WT (1, green), the antisense (2, dark gray) and the overexpression (3, light gray). The formation of clusters was mainly driven in the plane of PC1 (27.9%) and PC2 (15.9%), while PC3 (8.6%) was only responsible for a small separation of the data (Fig. 4c). A closer view of the factors driving the differentiation revealed high positive loadings of the total organic and amino acid counts, carbohydrates (glucose, fructose, sucrose and starch) and the individual organic acids citrate, lactate and isocitrate for PC1. Separation along PC2 was mainly driven by organic (succinate, aconitate and fumarate) and amino acids (proline and asparagine). Finally, PC3 separation was due to the amino acids glutamine, lysine and arginine (Fig. 4d; Table S4).

### Stomata count and size are unaffected, while GC starch showed a positive correlation with GDC‐H expression

To discover whether modified GC photorespiration caused alterations in stomatal properties (e.g. number and size), we analyzed epidermal peels by microscopy. As shown in Table 2, no significant changes were observed in stomatal density and index. Furthermore, stomata were invariant among all genotypes with regard to either length, width or area (Table 2).

**Table 2:** Overview of stomatal parameters of guard cell‐specific glycine decarboxylase H‐protein modulated lines and the wild‐type.

Genotype	Parameter				
	Stomatal density (mm^−2^)	Long axis (μm)	Short axis (μm)	Stomatal area (μm^−2^)	Stomatal index (mm^2^ mm^−2^)
SL1	142.48 ± 8.85^a^	20.99 ± 1.40^a^	14.66 ± 1.48^a^	966.22 ± 144.89^a^	0.14 ± 0.01^a^
SL4	157.76 ± 19.56^a^	21.13 ± 1.51^a^	14.68 ± 0.61^a^	975.77 ± 90.72^a^	0.15 ± 0.02^a^
SL7	167.14 ± 32.48^a^	20.04 ± 1.47^a^	13.72 ± 0.78^a^	864.41 ± 79.84^a^	0.14 ± 0.03^a^
Col.0	148.10 ± 20.30^a^	20.34 ± 0.63^a^	14.33 ± 1.53^a^	917.31 ± 106.78^a^	0.13 ± 0.01^a^
AL4	156.03 ± 27.80^a^	20.22 ± 1.88^a^	13.82 ± 1.43^a^	882.94 ± 143.41^a^	0.14 ± 0.02^a^
AL5	168.72 ± 37.97^a^	20.65 ± 1.24^a^	14.08 ± 0.77^a^	913.27 ± 67.03^a^	0.15 ± 0.04^a^
AL9	155.31 ± 12.84^a^	20.30 ± 0.93^a^	14.25 ± 0.93^a^	912.00 ± 124.10^a^	0.14 ± 0.01^a^

For microscopic determination of stomatal parameters, we used Arabidopsis plants grown under standard conditions to growth stage 5.1 (Boyes *et al*., 2001). Given are stomatal density, stomatal length, stomatal width, stomatal area and stomatal index. Shown are means ± SD of at least four biological replicates, with measurements of at least 10 stomata per leaf (40 stomata in total). Values that do not share the same letter are significantly different from each other as determined by ANOVA. Values in bold indicate *P* < 0.05.

**Fig. 5:**
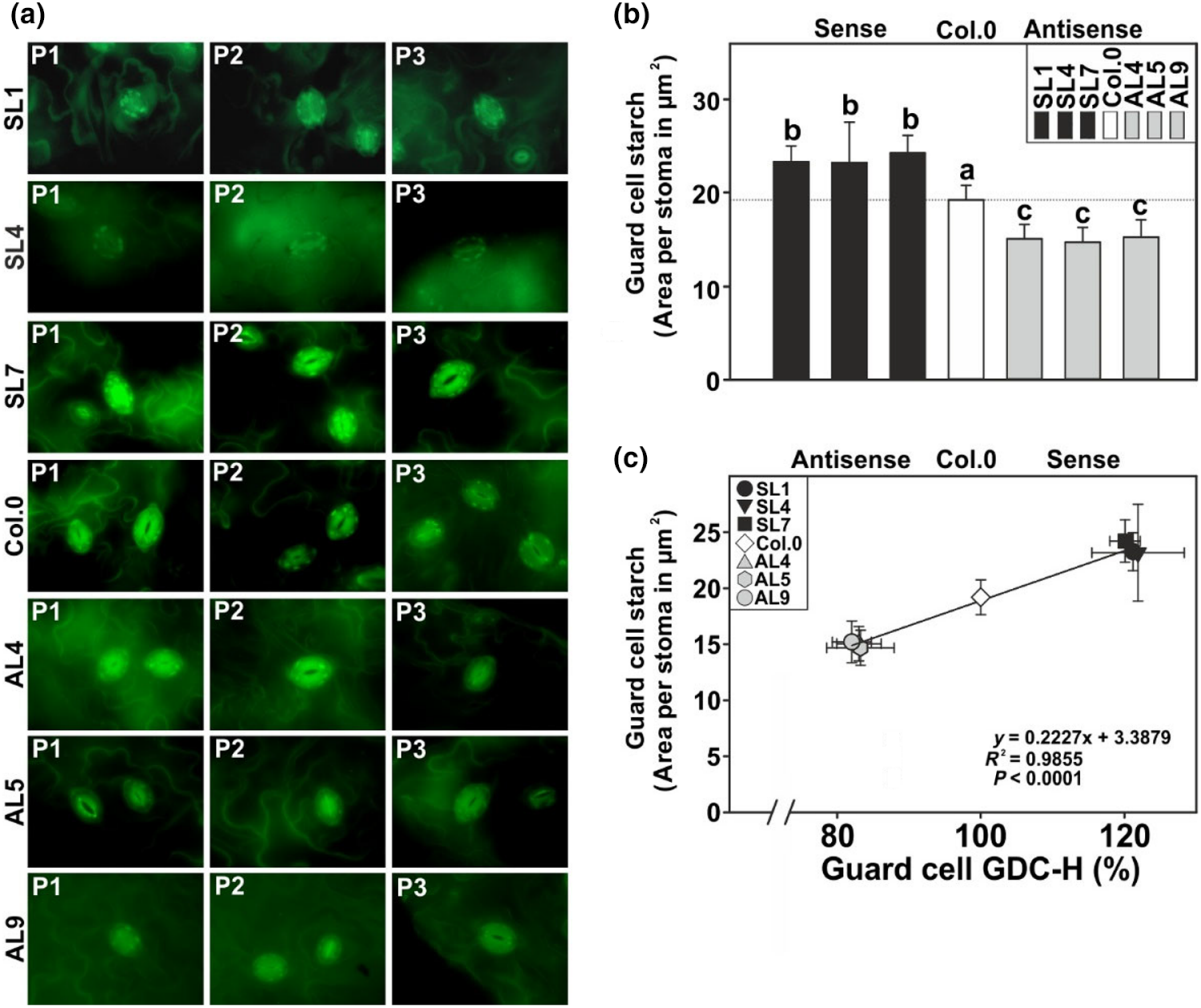
Guard cell (GC) starch quantification in GC‐specific glycine decarboxylase H‐protein (GDC‐H) mutant lines and the wild‐type (WT). For microscopic determination of GC starch accumulation patterns, we used Arabidopsis plants grown under standard conditions to growth stage 5.1 (Boyes *et al*., 2001). Given are (a) three representative microscopic images of each genotype following starch staining, (b) GC starch accumulation and (c) correlation analysis between GC GDC‐H protein expression and GC starch contents. Shown are means ± SD of at least four biological replicates, with at least 10 stomata per leaf (∑ > 40). Values that do not share the same letter are significantly different from each other as determined by ANOVA.

Given previous research revealed that GC starch amounts are key for its metabolism, that is rapid movements of stomata (Flütsch *et al*., 2020; Dang *et al*., 2024), we also analyzed the starch content of GC (Fig. 5a) in the same plant material. Interestingly, we observed a positive correlation between the GDC‐H expression and starch amounts in GC. More precisely, all the overexpression lines were significantly increased (up to 26.1%), while the antisense suppressors were decreased (up to −23.4%) in GC starch at the middle of the day (Fig. 5b,c). Collectively, the data suggest that stomatal aperture and their starch content, but not their size and amount, responded to the manipulation of GC photorespiration.

## Discussion

The important role of photorespiration in enabling efficient photosynthesis at atmospheric O_2_ levels is generally accepted (Bauwe *et al*., 2012; Busch, 2020). However, specific implications for stomatal behavior, especially GC metabolism, remained unexplored due to the absence of GC‐specific mutants (Lemonnier & Lawson, 2024). Earlier work showed that Arabidopsis WT plants shifted from high to low CO_2_, to induce photorespiratory activity, responded with only a few transcriptional changes, which were mainly genes related to drought stress. This is likely a consequence of reduced CO_2_ availability, prompting enhanced stomatal opening and potentially increased water loss (Eisenhut *et al*., 2017). Interestingly, when MC photorespiration is genetically impaired, this response pattern is abolished. Such mutants display reduced stomatal conductance (*g*_s_), transpiration rates and stronger transcriptional reprogramming than in WT plants. These changes included a marked reduction in phototropin (*PHOT1* and *PHOT2*) expression, which are essential for light‐dependent stomatal opening (Eisenhut *et al*., 2017) and, in particular, the species‐specific stomatal blue light response (Vialet‐Chabrand *et al*., 2021). Collectively, these findings pointed us to the hypothesis that active photorespiration is required for stomatal movements in response to changes in external CO_2_. As earlier work provided evidence for CO_2_ fixation and photorespiration in GC (Reckmann *et al*., 1990; Cardon & Berry, 1992; Lemonnier and Lawson, 2024), there is a need for further research to unravel potential interactions between photosynthesis, photorespiration and stomatal behavior. Moreover, we speculated that MC photorespiration may directly affect GC metabolism and, possibly vice versa, via contributing to the *C*_i_‐dependent regulatory mechanism coordinating *g*_*s*_ and photosynthesis.

Through GC‐specific genetic manipulation of the key photorespiratory enzyme GDC‐H (Timm *et al*., 2012a), we obtained indications that its function associated with photorespiration is required in GC and serves as a yet‐unidentified component contributing to optimal stomatal movements and metabolism in Arabidopsis leaves in current atmospheric air. This conclusion aligns with our observation that GC‐specific manipulation of *GDC‐H* expression impacts the phenotype and biomass accumulation (Figs 1, S2). The specificity of this manipulation, that is altered expression only in GC (Figs 1, S1), supports the previously raised hypothesis that photorespiration could be active in GC and demonstrates its role in supporting stomata functions. It is noteworthy stating biomass changes were most likely attributable to altered photorespiratory fluxes in GC which, in turn, impact stomatal behavior. This agrees with the fact that transgenic plants were visually indistinguishable under high CO_2_ conditions, suppressing photorespiration (Fig. S2A), and therefore minimizing any significant impact of photorespiratory manipulations. The statement is consistent with observations resulting from studies on classic photorespiratory mutants, which are lethal in normal air but fully recoverable in high CO_2_ on the phenotypical and physiological level (Somerville, 2001; Timm *et al*., 2012b; Eisenhut *et al*., 2017). However, although this growth behavior suggests the modulation of GDC‐H in GC mainly translates to altered photorespiratory fluxes in these cells, future work is required to fully resolve that this is indeed the case. Furthermore, biomass alterations in GC (this study) and whole leaf (e.g. Queval *et al*., 2007) photorespiratory mutants were pronounced under longer days (Fig. S2), which can be explained by the longer necessity for increased photorespiratory capacity with extended illumination, during which more toxic pathway intermediates potentially accumulate. Therefore, genetic modifications to the photorespiratory pathway exert a greater impact on plants grown under long‐day conditions.

Our photosynthetic characterization revealed that GC‐specific manipulation of photorespiratory GDC did not significantly affect MC photosynthetic light reactions (Fig. S3), as expected. However, GC‐specific overexpression of *GDC‐H* stimulated net CO_2_ assimilation rates (*A*_N_) across a wide range of light intensities (Fig. 2a) driven by increasing *g*_s_ and the removal of diffusional constraints on CO_2_ uptake indicated by the greater intracellular CO_2_ concentrations (*C*_i_) (Fig. 2b–e). Notably, such physiological responses occurred in the absence of alterations in stomatal amount and size (Table 2). This is in line with other studies that have manipulated GC, positively affecting *g*_s_ and *A*_N_, with unchanged stomatal characteristics (Wang *et al*., 2014). Notwithstanding, our findings suggest a direct correlation between GC GDC‐H protein expression, probably altering the photorespiratory flux, and *g*_s_. Mechanistically, this response could result from changes in photorespiratory metabolism, signaling varying CO_2_ availability within GC, which induces changes in aperture to supply more or less CO_2_ for mesophyll photosynthesis. Enhanced mesophyll photosynthesis due to greater *g*_s_ in the *GDC‐H* overexpression lines supports greater carbohydrate biosynthesis, stimulating growth. Both improved photosynthesis and increased accumulation of photosynthates (e.g. 3PGA, sucrose and transitory starch), alongside enhanced growth, were observed in these lines. Conversely, antisense suppression of *GDC‐H* led to the opposite effects (Figs 1, 2, 3, 4; Table 1). Similar outcomes have been reported for leaf‐specific manipulations of photorespiratory genes, including *GDC* (H‐ and L‐protein) and *PGLP1* (Timm *et al*., 2012a, 2015; Flügel *et al*., 2017; Simkin *et al*., 2017; López‐Calcagno *et al*., 2019). These effects were rationally explained by alleviating negative feedback inhibition of photorespiratory intermediates on the CB cycle, thereby improving RuBP regeneration and enhancing carbon assimilation and export (Bauwe, 2018; Timm & Hagemann, 2020). However, since gene expression driven by promoters such as *ST*‐*LSI* or *35S* is not fully restricted to MC, it remains uncertain whether the observed effects in these studies result exclusively from MC‐specific manipulation of photorespiration or whether GC photorespiratory metabolism and behavior have also contributed toward those changes. Our findings that GC‐specific manipulation of photorespiratory GDC plays a significant role in the regulation of stomatal movements could be due to the lowering of metabolic inhibitions on the CB cycle directly in GC and improved CO_2_ fixation in these cells that provides osmotic support for increased aperture. The improved photosynthesis in the overexpressors could potentially signal a higher GC CO_2_ demand (i.e. lowered *C*_i_), prompting stomata to open, with the opposite occurring in the antisense lines, characterized by lower photorespiratory flux and impaired CB cycle activity. Alternatively, the changes in metabolic fluxes in both photorespiration and photosynthesis in the GC could also influence GC electron transport rates (Lawson *et al*., 2002, 2003), which in turn would alter the redox state of the PQ pool, which has been suggested to play a role in regulating *g*_*s*_ in line with mesophyll photosynthetic demands (Lawson *et al*., 2008; Busch, 2014; Kromdijk *et al*., 2020; Taylor *et al*., 2024). In addition to examining light acclimation of photosynthesis, photosynthetic parameters in response to changes in external O_2_ concentrations were profiled, primarily to manipulate leaf photorespiration on a short‐term basis. Notably, our measurements revealed that GC‐specific manipulation of GDC significantly influenced the O_2_ susceptibility of the transgenic plants (Fig. 3). Specifically, increasing the GDC‐H expression in GC reduced the inhibitory effects of high O_2_ on photosynthesis, likely by mitigating the impact of increased RuBisCO oxygenation due to improved CO_2_ availability via greater *g*_s_ (Fig. 3c,f). Conversely, reducing the GDC‐H expression resulted in impaired photosynthetic parameters under the same CO_2_ : O_2_ ratios. It is worth highlighting that differences in photosynthetic parameters were negligible under low O_2_ conditions, suggesting the manipulation of GC GDC‐H amounts mainly affects photorespiration. This finding is consistent with the normalization of growth observed under elevated CO_2_, conditions that drastically diminish the need for efficient photorespiratory metabolism (Fig. 3). Furthermore, our findings agree with recent studies that highlight the utility of altered O_2_ levels as a proxy for modulating photorespiratory flux, even in the absence of genetic perturbations to the pathway (Fu *et al*., 2023; Smith *et al*., 2024).

Alongside specific changes in leaf‐carbohydrate accumulation patterns (Table 1), we observed only a few metabolic shifts in the transgenic lines through LC‐MS/MS analysis. These measurements revealed that plants mainly responded with a general increase in soluble amino acids and a decrease in organic acids following the GC‐specific upregulation of GDC‐H, with the opposite holding true for antisense suppressors (Fig. 4). Hence, it seems there is a correlation between elevated photosynthesis and the increased availability of carbon building blocks that are potentially used to support higher nitrogen assimilation rates to support amino acid biosynthesis to facilitate protein biosynthesis and increased plant growth (Figs 1, S2). This hypothesis is in line with experimental support from other research that pioneered a correlation between the photorespiratory flux, adjusted by external CO_2_ availability, and nitrogen assimilation (Rachmilevitch *et al*., 2004; Bloom *et al*., 2010). Alternatively, the increased amino acid‐to‐organic acid ratio can be explained by higher ammonia release in response to GDC activity upregulation.

Finally, and more importantly, simultaneous to the higher whole leaf‐carbohydrate status in the overexpressors (Table 1), we determined increased starch accumulation in GCs of the overexpressors, while lower amounts were present in the antisense mutants (Fig. 5). This observation could also explain altered stomatal behavior in the transgenic lines, as GC starch amount was reported to be of high importance for stomatal movements (Flütsch *et al*., 2020; Dang *et al*., 2024). Based on their findings, the authors suggest a higher availability in GC starch results in improved glucose availability, supporting rapid stomatal movements (Flütsch *et al*., 2020). Nevertheless, it remains an open question whether starch synthesis in GC is directly affected in response to GC‐specific GDC‐H manipulations or whether this is a result of the increased MC photosynthesis and carbohydrate biosynthesis. The latter argument is in favor of the general assumption that GC starch is mainly synthesized using carbon building blocks imported from the MC and further agrees with research on Arabidopsis sucrose synthases (Daloso *et al*., 2016; Piro *et al*., 2023).

**Fig. 6:**
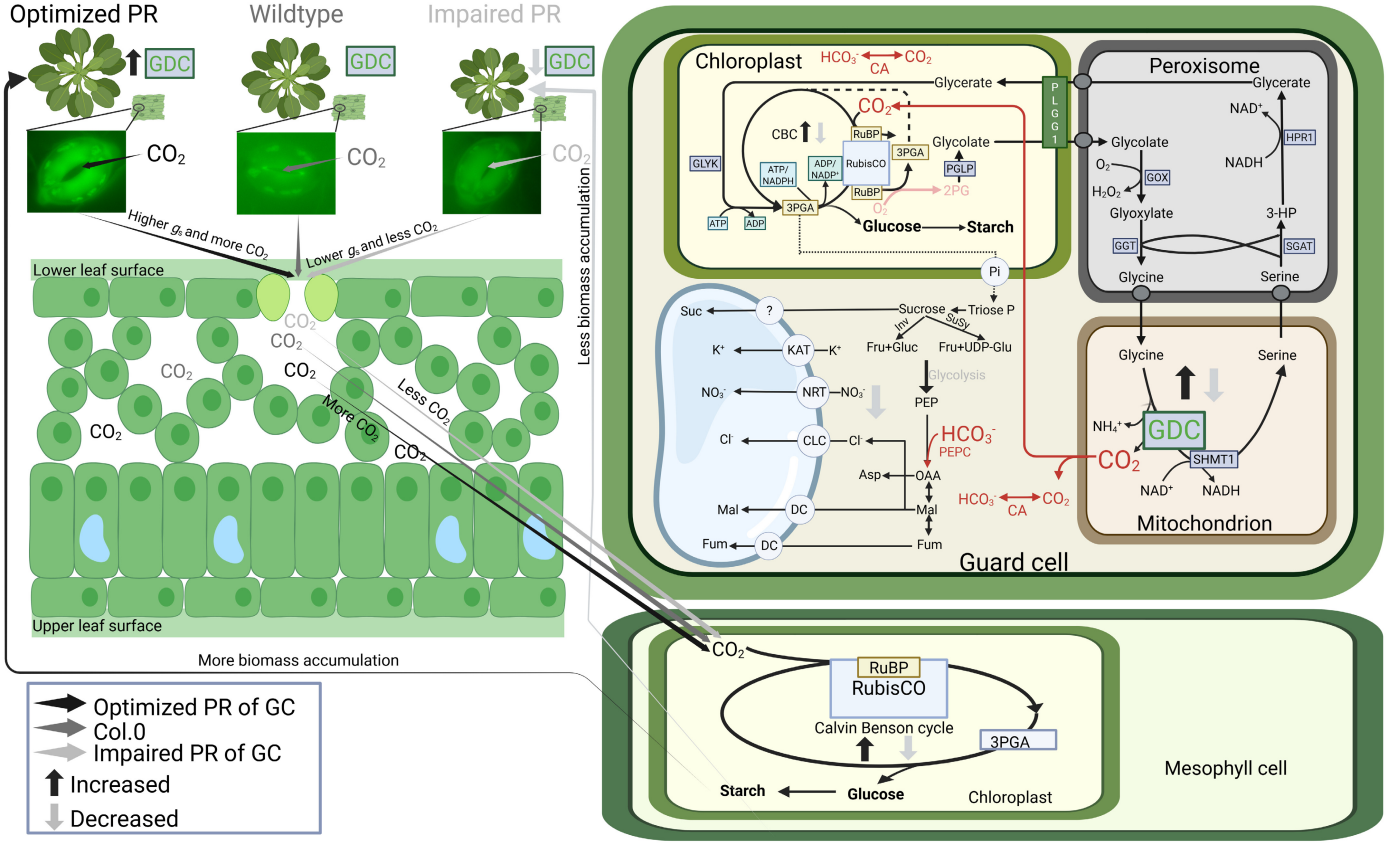
Scheme of current hypothesis on the impact of altered guard cell (GC) glycine decarboxylase H‐protein (GDC‐H) expression on stomatal movements in Arabidopsis. Stomata physiologically respond to changes in external CO_2_ availability, in turn affecting internal CO_2_ (*C*_i_). High CO_2_ leads to stomatal closure, while low CO_2_ leads to stomatal opening. We hypothesize that changes in *C*_i_, and the resulting movements, can be mimicked through our transgenic intervention, assumed to alter GC photorespiratory metabolism (i.e. GDC‐H protein expression). On the one hand, low external CO_2_ stimulates photorespiratory metabolism (more RuBisCO oxygenation), which is similar in the *GDC‐H* overexpression plants, and prompts stomata to be more open (low *C*_i_). On the other hand, high external CO_2_ suppresses photorespiratory metabolism (less RuBisCO oxygenation), which is comparable with our GC *GDC‐H* antisense lines, causing stomata to be more closed (high *C*_i_). Mechanistically, improved or lower (impaired) GC photorespiratory metabolism is hypothesized to alleviate or enhance negative metabolic feedback onto the Calvin–Benson (CB) cycle, improving or hindering RuBP regeneration. Improved CB cycle performance lowers *C*_i_ and makes GC a stronger CO_2_ sink, enhances stomatal opening (higher *g*_s_), to ultimately provide more CO_2_ for mesophyll photosynthesis. Higher photosynthesis stimulates carbohydrate biosynthesis and plant growth. Impaired or repressed CB cycle performance lowers CO_2_ requirements, leads to less open stomata (lower *g*_s_) and decreases mesophyll CO_2_. Reduced mesophyll photosynthesis provides less carbon for plant growth. However, given experimental evidence revealed only low RuBisCO amounts and CB cycle activity in GC, this hypothesis is only partially sufficient to explain the physiological responses. Thus, we further assume that manipulation of GC GDC activity (glycine decarboxylation) through increased and suppressed GDC‐H protein expression has a direct impact on the GC *C*_i_, too. More GDC activity (in the overexpression lines, increased glycine decarboxylation) leads to increased, while lower GDC activity (in antisense lines, reduced glycine decarboxylation) leads to reduced GC *C*_i_. Changes in GC *C*_i_ supports (or reduces) photosynthesis for increased (or decreased) GC starch production, providing more (or less) energy supply for stomatal movements. Changes in CO_2_ are likely to affect PEPC mediated GC photosynthesis. It seems also reasonable to consider an impact on GC nitrate and hydrogen peroxide amounts, as a consequence of altered GDC activity and photorespiration, as additional factors leading to changes in GC metabolism and stomatal movements. The figure was created with BioRender (https://BioRender.com/b29k163). Enzymes: CA, carbonic anhydrase; Inv, invertase; PEPc, phospho*enol*pyruvate carboxylase; PEPk, phospho*enol*pyruvate carboxykinase; RuBisCO, ribulose‐1,5‐bisphosphate carboxylase/oxygenase; SuSy, sucrose synthase. Metabolites: 3PGA, 3 phosphoglycerate; Asp, aspartate; Cl^−^, chloride; Fru, fructose; Fum, fumarate; Glu, glutamate; Gluc, glucose; Isoc, isocitrate; K^+^, potassium; Mal, malate; NO_3_^−^, nitrate; OAA, oxaloacetate; PEP, phospho*enol*pyruvate; Suc, sucrose; UDP‐glu, uridine diphosphate glucose. Transporters: CLC, chloride channel; DC, dicarboxylate carrier; NRT, nitrate transporter; OC, putative oxaloacetate carrier.

The findings of this study are summarized in the tentative model illustrated in Fig. 6. This model proposes that changes in external CO_2_ availability are most likely detected and translated by the GC photorespiratory flux in response to alterations in *C*_*i*_, which modulates photosynthetic activities, electron transport or an other unknown metabolic process within these specialized cells associated with GDC activity during illumination. Consequently, stomatal movements are adjusted according to the availability of energy resources, including the amounts of GC starch (Fig. 5) and/or sensing changes in CO_2_ to which they respond. Mechanistically, variations in photorespiratory GDC activity could influence CB cycle activities by alleviating or intensifying negative metabolic feedback on the regenerative branch of the pathway and altering the extent of carbon export from the cycle. This hypothesis is based on experimental evidence from studies manipulating photorespiratory flux in MC (Timm *et al*., 2012a, 2015; Flügel *et al*., 2017). However, given that GCs contain only a few chloroplasts (Allaway & Setterfield, 1972; Willmer & Fricker, 1996) and as such have comparably low rates of RuBisCO‐mediated CO_2_ fixation, it seems likely to assume this hypothesis is not fully sufficient to explain the phenotypic and physiological alterations observed in the GC‐specific transgenic plants. Possibly, the observed stimulations result more directly from changes in mitochondrial photorespiratory metabolism associated with GDC. Hence, we hypothesize that changes in GC GDC‐H expression particularly affect glycine decarboxylation, directly impacting GC's *C*_i_, thereby influencing photosynthetic activities. On the one hand, the surplus CO_2_ released from mitochondria can facilitate the reduction in the oxygenation reaction of RuBisCO and stimulate the CB cycle (Fig. 6). As discussed earlier, we assume this is rather unlikely because of the low GC RuBisCO and chloroplast content (Humble & Raschke, 1971; Vaughn, 1987; Willmer & Fricker, 1996). On the other hand, the increase in mitochondrially driven CO_2_ could, at least partially, be converted to bicarbonate via carbonic anhydrase, facilitating PEPC‐mediated CO_2_ fixation in GC (Tarczynski & Outlaw Jr, 1990). This process would result in increased energy resources to drive stomatal movements. Other possibilities of how potential changes in GC photorespiratory metabolism could alter stomatal behavior might involve GC nitrate or H_2_O_2_ amounts, two molecules known to influence stomatal movements (Guo *et al*., 2003; Shi *et al*., 2024), as both respond directly or indirectly by photorespiration. Further work is essentially required to fully elucidate the exact mechanism by which changes in GDC activity specifically in GC influence stomatal behavior. Such approaches should involve other photorespiratory enzymes, in order to get more direct evidence if the observed findings are specific for GDC, and mutants with parallel modulation of GC‐modulated PEPC.

### Conclusion and outlook

Based on the findings on the GC‐specific manipulation of GDC‐H, we propose that photorespiration, that is mitochondrial photorespiratory metabolism associated with GDC, has new implications for the C3 plant Arabidopsis. It seems reasonable to assume that GC photorespiration might be involved in the regulation of optimal stomatal behavior, including *g*_s_. This metabolic interplay could represent a prerequisite to adapt to variations in the external CO_2_ : O_2_ ratios that affect *C*_i_ (Fig. 6). We currently hypothesize that changes in GC photorespiratory flux could eventually signal the GC CO_2_ demands in response to external CO_2_ availability, in turn communicating with the mesophyll to adjust mesophyll photosynthesis and carbohydrate biosynthesis. However, how alterations in the GC photorespiratory flux are sensed and incorporated into the CO_2_ sensing and signaling cascade remains an open question. In order to resolve this regulation circuit in more detail, future studies on MC‐ and GC‐specific photorespiratory manipulations are required. These should involve additional enzymes, located in different subcellular compartments, and a GC CO_2_ signaling mutant background.

## Competing interests

None declared.

## Author contributions

ST conceived and supervised the project. HS and ST designed the research. NS performed cloning procedures and established the transgenic lines. HS performed the research. HS, TL, MH and ST analyzed the data. MH provided experimental equipment and tools. ST wrote the article, with additions and revisions from HS, TL and MH. All authors have read and approved the final version of the manuscript.

## Disclaimer

The New Phytologist Foundation remains neutral with regard to jurisdictional claims in maps and in any institutional affiliations.

## Supporting information


**Fig. S1** Generation and verification of Arabidopsis guard cell‐specific glycine decarboxylase H‐protein overexpression and antisense lines.
**Fig. S2** Phenotype of Arabidopsis guard cell‐specific glycine decarboxylase H‐protein modulated lines and the wild‐type under different growth conditions.
**Fig. S3** Chlorophyll fluorescence parameters of Arabidopsis guard cell‐specific glycine decarboxylase H‐protein modulated lines and the wild‐type.
**Table S1** Light–response curves of the transgenic lines and the wild‐type under standard conditions.
**Table S2** Calculated parameters from light–response curves numerically given in Table S1.
**Table S3** Abundances of selected intermediates associated with primary metabolism in the transgenic lines and the wild‐type under standard conditions.
**Table S4** Loadings of metabolites on the first three principal components in leaves of the guard cell‐specific glycine decarboxylase H‐protein lines and the wild‐type.
**Table S5** Primers used for PCR amplification of genomic DNA and cDNA.
Please note: Wiley is not responsible for the content or functionality of any Supporting Information supplied by the authors. Any queries (other than missing material) should be directed to the *New Phytologist* Central Office.

## Data Availability

The data supporting the findings of this study are presented in Figs 1, 2, 3, 4, 5 and Table 1 included in the main text and Supplemental Data (Figs S1–S3; Tables S1–S4) associated with this article. Plasmids and transgenic plants generated in this study will be made available upon request to the corresponding author. Accession no.; EMBL sequence data bank no.: Z25856.
