# Supplementary material for: Guard cell‐specific glycine decarboxylase manipulation affects Arabidopsis photosynthesis, growth and stomatal behavior
Source: New Phytol. 2025 Apr 11;246(5):2102–17. doi: 10.1111/nph.70124 (PMC12059538; doi:10.1111/nph.70124)
Supplement: Supplementary file 1 — Fig. S1 Generation and verification of Arabidopsis guard cell‐specific glycine decarboxylase H‐protein overexpression and antisense lines. Fig. S2 Phenotype of Arabidopsis guard cell‐specific glycine decarboxylase H‐protein modulated lines and the wild‐type under different growth conditions. Fig. S3 Chlorophyll fluorescence parameters of Arabidopsis guard cell‐specific glycine decarboxylase H‐protein modulated lines and the wild‐type. Table S1 Light–response curves of the transgenic lines and the wild‐type under standard conditions. Table S2 Calculated parameters from light–response curves numerically given in Table S1. Table S3 Abundances of selected intermediates associated with primary metabolism in the transgenic lines and the wild‐type under standard conditions. Table S4 Loadings of metabolites on the first three principal components in leaves of the guard cell‐specific glycine decarboxylase H‐protein lines and the wild‐type. Table S5 Primers used for PCR amplification of genomic DNA and cDNA. Please note: Wiley is not responsible for the content or functionality of any Supporting Information supplied by the authors. Any queries (other than missing material) should be directed to the New Phytologist Central Office. [file NPH-246-2102-s001.docx]

## *New Phytologist* Supporting Information

Article title: **Guard-cell-specific glycine decarboxylase manipulation affects Arabidopsis photosynthesis, growth and stomatal behavior**

Authors: Hu Sun, Nils Schmidt, Tracy Lawson, Martin Hagemann, Stefan Timm

Article acceptance date: 15 March 2025

The following Supporting Information is available for this article:

**Fig. S1** Generation and verification of Arabidopsis GC specific *GDC-H* overexpression and antisense lines.

**Fig. S2** Phenotype of Arabidopsis GC specific *GDC-H* modulated lines and the wildtype under different growth conditions.

**Fig. S3** Chlorophyll fluorescence parameters of Arabidopsis GC specific *GDC-H* modulated lines and the wildtype.

**Table S1** Light response curves of the transgenic lines and the wildtype under standard conditions.

**Table S2** Calculated parameters from light response curves numerically given in Suppplemental Table S1.

**Table S3** Abundances of selected intermediates associated with primary metabolism in the transgenic lines and the wildtype under standard conditions.

**Table S4** Loadings of metabolites on the first three principal components (PCs) in leaves of the GC specific *GDC-H* lines and the wildtype.

**Table S5** Primers used for PCR amplification of genomic DNA and cDNA.

**Fig. S1** Schematic overview of the GC specific *FpGDC-H* **(A)** overexpression and **(B)** antisense constructs. **(C)** PCR verification of the transformed constructs into the genome of transgenic **(C1)** overexpression and **(C2)** antisense lines and the corresponding loading control **(C3)**. **(D)** RT-PCR verification of the full-length *FpGDCH* **(D1)** and the *AtGDCH1* **(D2)** transcripts, in comparison with signals of the constitutively expressed 40S ribosomal protein *S16* gene as loading control **(D3)**. **(E)** Immunoblots of protein extracts from **(E1)** MC, **(E2)** GC and whole leaves **(E3)** using a specific antibody against mitochondrial GDC-H (Timm et al., 2013) and chloroplastidal 2-phosphoglycolate phosphatase 1 (PGLP1; Flügel et al., 2017) as loading control. Note, loading control immunoblots were essentially developed from the same membrane as for GDC-H.


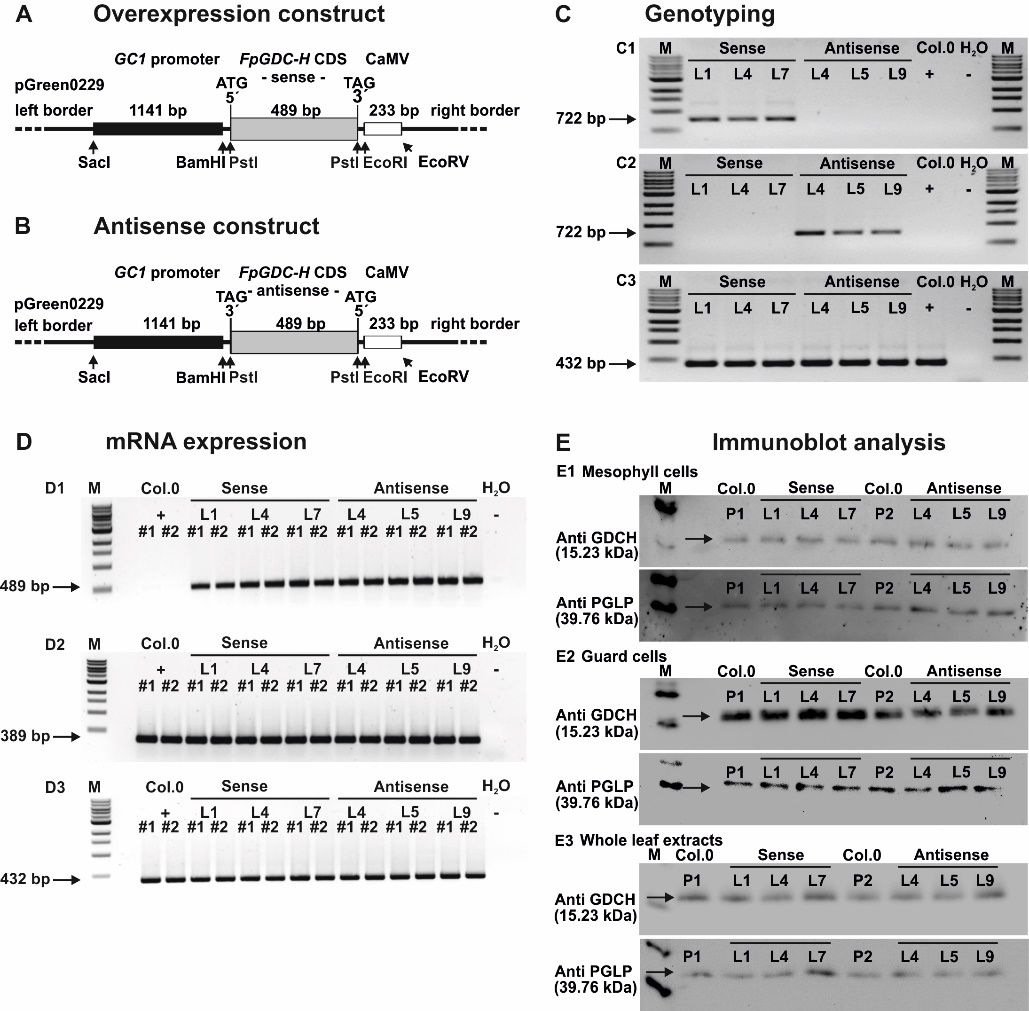


**Fig. S2 (A)** Representative images of plants grown for 4 weeks in normal air (upper panel: LC – low carbon; 400 ppm CO_2_) or in elevated CO_2_ (lower panel: HC – low carbon; 3000 ppm CO_2_) in a 12/12 h day-/night-cycle. Representative images of plants grown for 4 weeks in normal air (LC – low carbon; 400 ppm CO_2_) in a **(B)** 10/14 h and **(C)** 14/10 h day-/night-cycle with otherwise equal conditions.


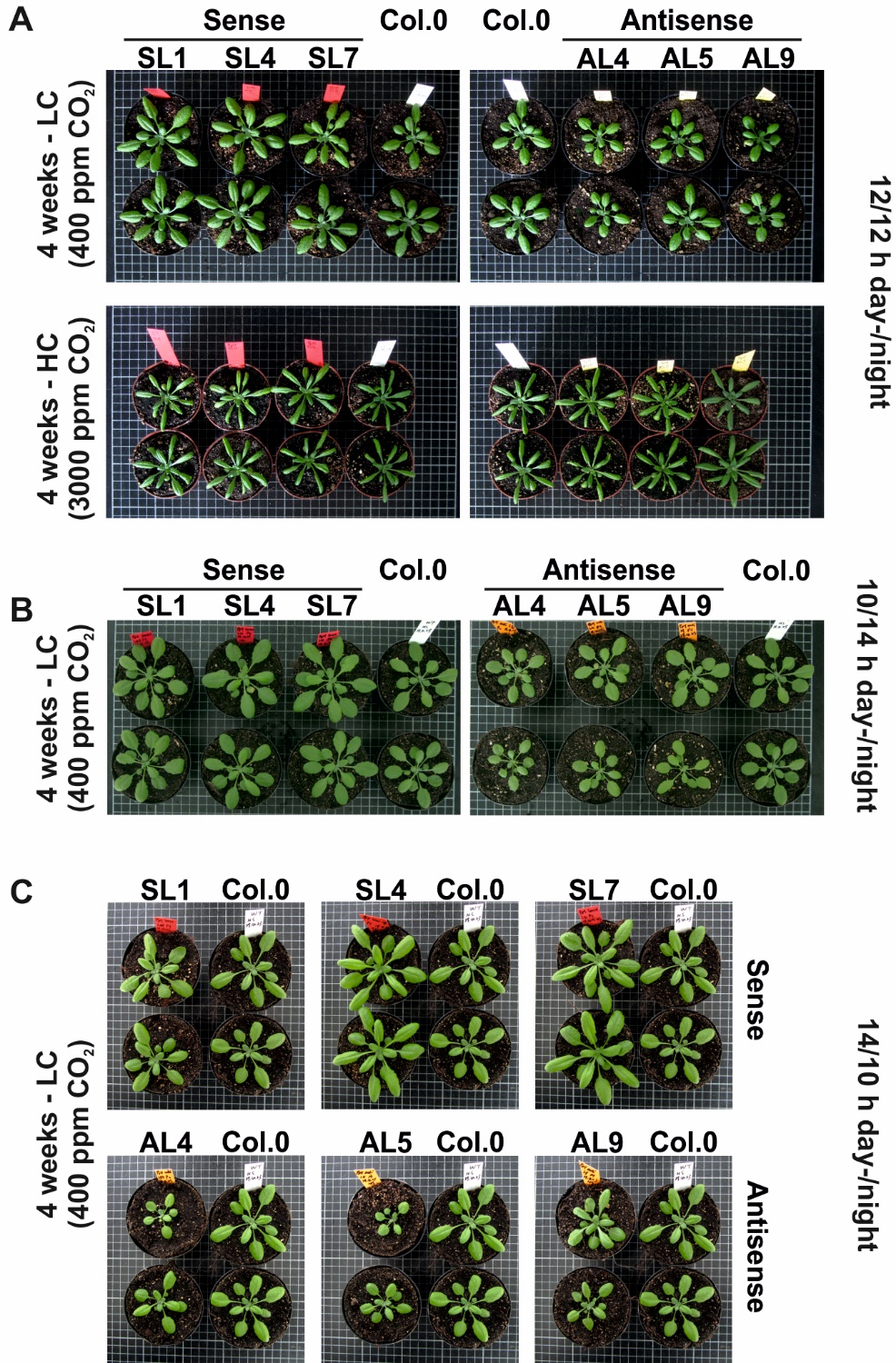


**Fig. S3** Displayed are selected parameters associated with PSII and PSI integrity and functioning of all genotypes grown under standard growth conditions to stage 5.1 (Boyes et al., 2001). Given are: **(A)** Maximum efficiency of PSII (*F_v_*/*F_m_*) and maximum oxidizable P700 (*P_m_*) from dark adapted plants; **(B)** photosynthetic efficiency curves of PSII (Y[II]) and PSI (Y[I]); **(C)** relative electron transport rates of PSII (rETR II) and PSI (rETR I); **(D)** non-photochemical quenching of PSII (NPQ) and cyclic electron flow around PSI (CET); and, **(E)** acceptor (Y [NA]) and donor (Y [ND]) side limitation of PSI. Shown are means ± SD of at least 6 biological replicates. Values that do not share the same letter are significantly different from each other as determined by ANOVA. Note, lack of letters in B to E is explained due to absence of statistical differences.


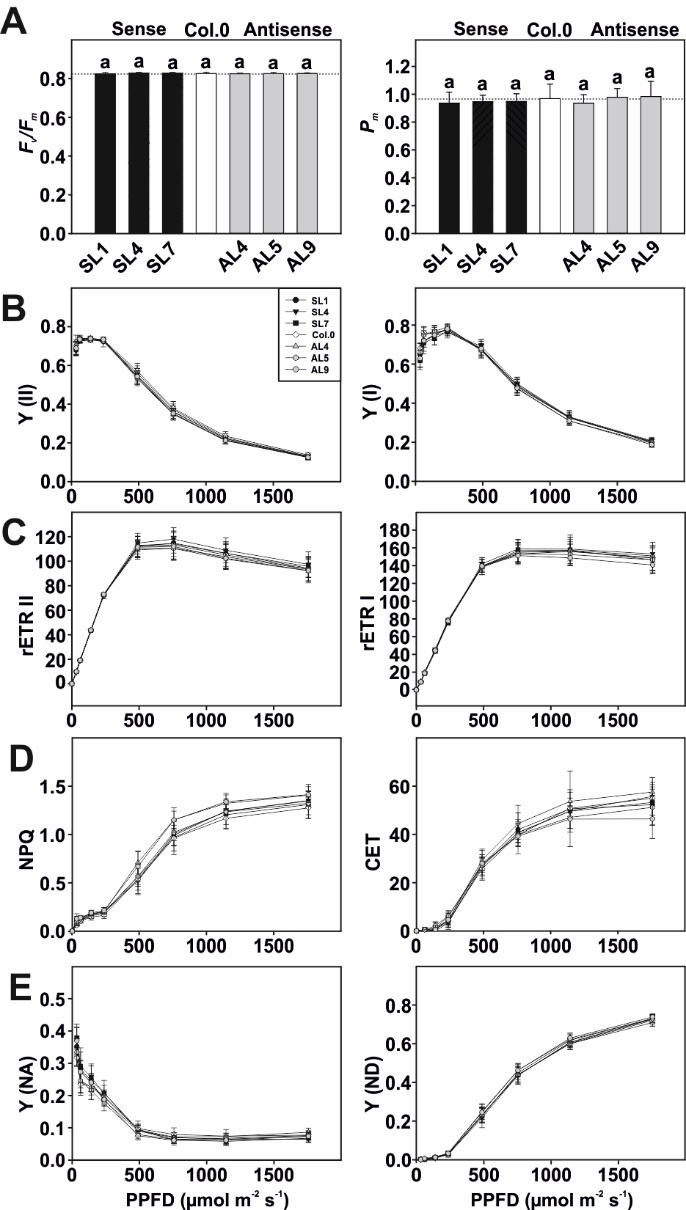


**Table S1** Plants were grown under environmental controlled conditions in normal air (400 ppm CO2) to growth stage 5.1 (Boyes et al., 2001). Light response curves were measured from high-to-low light (1759,1144,757,488,236,143,62,36, and 0 μmol m-2 s-1) following 10 min of light adaptation (1000 μmol m-2 s-1). Given are: net CO2 assimilation rate (AN), stomatal conductance (gs), intracellular CO2 concentration (Ci), transpiration rate (E); and intrinsic water use efficiency (WUE). Values are means ± SD (n = 6). Values that do not share the same letter are significantly different from each other as determined by ANOVA.

|  |  | **Light intensity (μmol m^-2^ s^-1^)** | | | | | | | | |
| --- | --- | --- | --- | --- | --- | --- | --- | --- | --- | --- |
|  | **Line** | **0** | **36** | **62** | **143** | **236** | **488** | **757** | **1144** | **1759** |
|  | SL1 | - 0.15 ± 0.60^a^ | 1.87 ± 0.48^a^ | 3.02 ± 0.53^a^ | 5.63 ± 6.98^a^ | 6.99 ± 0.86^a^ | 8.11 ± 0.76^b^ | 8.39 ± 0.69^b^ | 8.65 ± 0.73^b^ | 8.88 ± 0.40^b^ |
|  | SL4 | - 0.25 ± 0.91^a^ | 1.76 ± 1.11^a^ | 2.59 ± 1.34^ab^ | 5.64 ± 1.36^a^ | 7.15 ± 1.08^a^ | 8.48 ± 1.09^b^ | 8.76 ± 1.00^b^ | 8.84 ± 1.05^b^ | 9.08 ± 0.89^b^ |
| *A*_N_ | SL7 | - 0.1 ± 1.14^a^ | 2.25 ±0.99^a^ | 2.95 ± 1.12^ab^ | 6.07 ± 0.75^a^ | 7.40 ± 1.29^a^ | 8.57 ± 0.96^b^ | 9.03 ± 0.80^b^ | 9.33 ± 0.82^b^ | 9.19 ± 1.03^b^ |
| (μmol CO_2_ m^-2^ s^-1^) | Col.0 | - 0.16 ± 0.74^a^ | 1.82 ± 0.79^a^ | 2.62 ± 0.86^ab^ | 5.20 ± 0.29^a^ | 6.45 ± 0.44^a^ | 7.05 ± 0.61^a^ | 7.70 ± 0.63^a^ | 7.92 ± 0.25^a^ | 7.90 ± 0.44^a^ |
|  | AL4 | - 0.32 ± 0.49^a^ | 1.03 ± 0.25^a^ | 2.27 ± 0.51^b^ | 4.31 ± 0.34^b^ | 5.36 ± 0.50^b^ | 5.98 ± 0.51^c^ | 6.09 ± 0.73^c^ | 6.36 ± 0.36^c^ | 6.41 ± 0.34^c^ |
|  | AL5 | - 0.80 ± 0.44^a^ | 1.27 ± 0.16^a^ | 2.32 ± 0.16^b^ | 4.93 ± 0.69^ab^ | 5.88 ± 0.69^b^ | 6.57 ± 0.43^c^ | 6.69 ± 0.45^c^ | 7.16 ± 0.77^c^ | 7.15 ± 0.51^c^ |
|  | AL9 | - 0.83 ± 0.44^a^ | 1.68 ± 0.31^a^ | 1.98 ± 0.32^b^ | 4.49 ± 0.64^b^ | 5.53 ± 0.41^b^ | 6.35 ± 0.43^c^ | 6.42 ± 0.93^c^ | 6.60 ± 0.36^c^ | 6.80 ± 0.37^c^ |
|  | SL1 | 0.17 ± 0.07^a^ | 0.20 ± 0.07^a^ | 0.23 ± 0.07^a^ | 0.26 ± 0.06^b^ | 0.30 ± 0.05^b^ | 0.33 ± 0.05^b^ | 0.36 ± 0.05^b^ | 0.38 ± 0.05^b^ | 0.40 ± 0.05^b^ |
|  | SL4 | 0.18 ± 0.04^a^ | 0.21 ± 0.04^a^ | 0.24 ± 0.04^a^ | 0.28 ± 0.04^b^ | 0.30± 0.03^b^ | 0.33 ± 0.03^b^ | 0.35 ± 0.03^b^ | 0.37 ± 0.03^b^ | 0.39 ± 0.03^b^ |
| *g*_s_ | SL7 | 0.17 ± 0.03^a^ | 0.19 ± 0.04^a^ | 0.23 ± 0.04^a^ | 0.26 ± 0.04^b^ | 0.28 ± 0.04^b^ | 0.31 ± 0.04^b^ | 0.33 ± 0.04^b^ | 0.35 ± 0.04^b^ | 0.37 ± 0.03^b^ |
| (mol m^-2^ s^-1^) | Col.0 | 0.14 ± 0.03^a^ | 0.16 ± 0.03^a^ | 0.19 ± 0.03^a^ | 0.20 ± 0.03^a^ | 0.22 ± 0.03^a^ | 0.23 ± 0.03^a^ | 0.25 ± 0.03^a^ | 0.26 ± 0.02^a^ | 0.28 ± 0.03^a^ |
|  | AL4 | 0.08 ± 0.03^b^ | 0.09 ± 0.03^b^ | 0.11 ± 0.04^b^ | 0.13 ± 0.04^c^ | 0.14 ± 0.04^c^ | 0.16 ± 0.04^c^ | 0.17 ± 0.04^c^ | 0.19 ± 0.04^c^ | 0.21 ± 0.03^c^ |
|  | AL5 | 0.08 ± 0.02^b^ | 0.10 ± 0.02^b^ | 0.12 ± 0.03^b^ | 0.13 ± 0.03^c^ | 0.15 ± 0.03^c^ | 0.16 ± 0.03^c^ | 0.18 ± 0.03^c^ | 0.20 ± 0.02^c^ | 0.22 ± 0.02^c^ |
|  | AL9 | 0.06 ± 0.02^b^ | 0.07 ± 0.02^b^ | 0.08 ± 0.03^b^ | 0.10 ± 0.03^c^ | 0.11 ± 0.03^c^ | 0.12 ± 0.04^c^ | 0.14 ± 0.04^c^ | 0.16 ± 0.04^c^ | 0.19 ± 0.04^c^ |
|  | SL1 | 394.1 ± 8.1^a^ | 378.3 ± 7.3^b^ | 371.4 ± 3.6^b^ | 355.3± 4.7^b^ | 351.4 ± 4.5^b^ | 345.6 ± 6.0^b^ | 346.6 ± 5.4^b^ | 346.9 ± 4.0^b^ | 346.9 ± 3.3^b^ |
|  | SL4 | 392.6 ± 8.1^a^ | 381.1 ± 8.9^b^ | 376.7 ± 6.04^b^ | 358.3 ± 4.5^b^ | 351.3 ± 3.6^b^ | 346.2 ± 2.7^b^ | 346.4± 2.1^b^ | 347.6 ± 3.0^b^ | 347.4 ± 1.9^b^ |
| *C*_i_ | SL7 | 390.5 ± 12.6^a^ | 377.6 ± 10.7^ab^ | 375.2 ± 3.29^b^ | 353.1 ± 1.0^b^ | 350.1 ± 4.5^b^ | 344.3 ± 2.5^b^ | 342.6± 3.1^b^ | 342.6 ± 1.2^b^ | 344.6± 2.4^ab^ |
| (μmol) | Col.0 | 393.1 ± 5.5^a^ | 372.3 ± 1.9^a^ | 367.0 ± 5.8^a^ | 346.2 ± 8.6^a^ | 339.1 ± 8.7^a^ | 337.6 ± 4.8^a^ | 335.8 ± 6.7^a^ | 336.6 ± 3.7^a^ | 338.8 ± 6.7^a^ |
|  | AL4 | 397.1 ± 12.4^a^ | 370.5 ± 9.1^a^ | 345.4 ± 12.2^c^ | 331.0 ± 12.6^a^ | 324.2± 13.5^c^ | 323.8 ± 9.6^c^ | 329.5 ± 9.3^c^ | 332.1 ± 8.7^ac^ | 336.7 ± 8.6^a^ |
|  | AL5 | 408.6 ± 7.8^a^ | 367.9 ± 8.9^a^ | 354.5 ± 14.2^c^ | 325.2 ± 17.9^c^ | 320.9 ± 17.4^c^ | 320.0 ± 13.6^c^ | 325.2 ± 10.2^c^ | 325.6 ± 11.1^c^ | 331.4 ± 8.1^ac^ |
|  | AL9 | 417.1± 13.9^a^ | 344.3 ± 20.2^c^ | 344.6 ± 25.4^c^ | 305.7 ± 20.5^c^ | 298.9 ± 34.1^c^ | 294.7 ±31.5^c^ | 306.5± 24.0^c^ | 318.7 ± 20.5^c^ | 324.6 ± 14.2^c^ |
|  | SL1 | 2.24 ± 0.56^a^ | 2.54 ± 0.55^a^ | 2.88 ± 0.52^a^ | 3.28 ± 0.53^a^ | 3.69 ± 0.57^b^ | 4.15 ± 0.60^b^ | 4.58 ± 0.63^b^ | 5.05 ± 0.64^b^ | 5.65 ± 0.65^b^ |
|  | SL4 | 2.30 ± 0.43^a^ | 2.59 ± 0.38^a^ | 2.93 ± 0.34^a^ | 3.29 ± 0.32^a^ | 3.61 ± 0.31^b^ | 4.00 ± 0.30^b^ | 4.31 ± 0.31^b^ | 4.73 ± 0.33^b^ | 5.18 ± 0.29^b^ |
| *E* | SL7 | 2.07 ± 0.31^a^ | 2.33 ± 0.33^a^ | 2.64 ± 0.34^a^ | 2.97 ± 0.34^a^ | 3.27 ± 0.34^b^ | 3.64 ± 0.32^b^ | 3.98 ± 0.33^b^ | 4.43 ± 0.39^b^ | 4.83 ± 0.33^b^ |
| (μmol H_2_O m^-2^ s^-1^) | Col.0 | 1.80 ± 0.39^a^ | 2.01 ± 0.41^a^ | 2.25 ± 0.37^a^ | 2.47 ± 0.39^a^ | 2.67 ± 0.38^a^ | 2.92 ± 0.35^a^ | 3.21 ± 0.35^a^ | 3.52 ± 0.35^a^ | 3.96 ± 0.40^a^ |
|  | AL4 | 1.01 ± 0.38^b^ | 1.17 ± 0.43^b^ | 1.41 ± 0.46^b^ | 1.61 ± 0.49^b^ | 1.82 ± 0.48^c^ | 2.08 ± 0.51^c^ | 2.36 ± 0.53^c^ | 2.68 ± 0.55^c^ | 3.05 ± 0.53^c^ |
|  | AL5 | 1.00 ± 0.27^b^ | 1.19 ± 0.29^b^ | 1.42 ± 0.34^b^ | 1.66 ± 0.34^b^ | 1.87 ± 0.34^c^ | 2.12 ± 0.33^c^ | 2.41 ± 0.30^c^ | 2.76 ± 0.23^c^ | 3.16 ± 0.19^c^ |
|  | AL9 | 0.68 ± 0.25^b^ | 0.81 ± 0.26^b^ | 1.01 ± 0.34b | 1.18 ± 0.37^b^ | 1.39 ± 0.41^c^ | 1.58 ± 0.50^c^ | 1.86 ± 0.56^c^ | 2.20 ± 0.55^c^ | 2.66 ± 0.55^c^ |
|  | SL1 | - 0.19 ± 0.39^a^ | 0.82 ± 0.26^a^ | 1.11 ± 0.22^a^ | 1.80 ± 0.29^a^ | 1.97 ± 0.27^b^ | 2.01 ± 0.27^b^ | 1.88 ± 0.23^b^ | 1.75 ± 0.20^b^ | 1.61 ± 0.20^b^ |
|  | SL4 | - 0.01 ± 0.47^a^ | 0.77 ± 0.51^a^ | 0.98 ± 0.52^a^ | 1.81 ± 0.48^a^ | 2.06 ± 0.42^ab^ | 2.20 ± 0.36^ab^ | 2.08 ± 0.32^ab^ | 1.92 ± 0.30^ab^ | 1.80 ± 0.23^ab^ |
| *WUE* | SL7 | - 0.16 ± 0.58^a^ | 1.04 ± 0.54^a^ | 1.20 ± 0.52^a^ | 2.09 ± 0.41^a^ | 2.30 ± 0.50^ab^ | 2.41 ± 0.35^ab^ | 2.31 ± 0.28^a^ | 2.14 ± 0.24^a^ | 1.94 ± 0.25^ab^ |
| (μmol CO_2_ mol^-1^ H_2_O) | Col.0 | - 0.03 ± 0.43^a^ | 0.95 ± 0.40^a^ | 1.17 ± 0.39^a^ | 2.22 ± 0.49^ab^ | 2.54 ± 0.47^ac^ | 2.49 ± 0.30^ac^ | 2.51 ± 0.40^a^ | 2.31 ± 0.27^a^ | 2.07 ± 0.35^ac^ |
|  | AL4 | - 0.41 ± 0.53^a^ | 0.98 ± 0.40^a^ | 1.59 ± 0.49^a^ | 2.73 ± 0.54^b^ | 3.00 ± 0.56^cd^ | 2.95 ± 0.42^c^ | 2.61 ± 0.38^ac^ | 2.45 ± 0.39^ac^ | 2.14 ± 0.36^ac^ |
|  | AL5 | - 0.80 ± 0.42^a^ | 1.10 ± 0.39^a^ | 1.72 ± 0.60^a^ | 3.01 ± 0.73^c^ | 3.21 ± 0.69^d^ | 3.19 ± 0.52^cd^ | 2.84 ± 0.38^cd^ | 2.63 ± 0.38^c^ | 2.30 ± 0.19^cd^ |
|  | AL9 | - 1.30 ± 0.58^a^ | 1.95 ± 0.82^a^ | 1.97 ± 0.86^a^ | 3.60 ± 0.75^c^ | 3.95 ± 0.98^d^ | 4.10 ± 1.04^d^ | 3.37 ± 0.44^d^ | 2.80 ± 0.40^c^ | 2.61 ± 0.44^d^ |

**Table S2** Estimations of the maximum photosynthetic rate (*A_max_*) and initial slopes of the light response curves (*α*_p_) from the light response curves measured from the transgenic lines in comparison with the wildtype. *A_max_* showed ~10-15% increases in overexpression and ~15-19% decreases in antisense lines. Values of *α*_p_ were accelerated to about 40% in overexpressors, but remained significantly unchanged in the antisense suppressors compared to the wildtype. Given are means ± SD (n = 6). Values that do not share the same letter are significantly different from each other as determined by ANOVA.

|  | Parameter |  |
| --- | --- | --- |
| Genotype | *A_max_* | *α*_p_ |
| SL1 | **8.98 ± 0.87^b^** | **0.14 ± 0.05^b^** |
| SL4 | **9.26 ± 0.86^b^** | **0.14 ± 0.01^b^** |
| SL7 | **9.40 ± 1.07^b^** | **0.13 ± 0.02^b^** |
| Col.0 | 8.15 ± 0.43^a^ | 0.09 ± 0.01^a^ |
| AL4 | **6.57 ± 0.64^c^** | 0.08 ± 0.01^a^ |
| AL5 | **7.05 ± 0.56^c^** | 0.08 ± 0.01^a^ |
| AL5 | **6.94 ± 0.39^c^** | 0.09 ± 0.01^a^ |

**Table S3** Plants were grown under environmental controlled conditions in normal air (400 ppm CO_2_) to growth stage 5.1 (Boyes et al., 2001). Leaf-material was harvested at the end of the day (11 h illumination) and frozen in liquid nitrogen until LC-MS/MS analysis. Given are absolute contents (nmol * mg DW^-1^) of 35 primary metabolites and the sum parameters of the total amino acid (AAs) and organic acid (OAs) amounts (both in µmol * mg DW^-1^). Values are mean ± SD from 6-8 biological replicates. Values that do not share the same letter are significantly different from each other as determined by ANOVA.

|  | **Genotype** | | | | | | |
| --- | --- | --- | --- | --- | --- | --- | --- |
| **Metabolite** | **SL1** | **SL4** | **SL7** | **Col.0** | **AL4** | **AL5** | **AL9** |
| 2PG | **196.81 ±14.20^b^** | **200.48 ± 16.75^b^** | **199.09 ± 4.81^b^** | 222.61 ±7.1^a^ | 236.6 ±19.77^a^ | 250.39 ± 9.14^a^ | 244.86 ± 22.09^a^ |
| 3PGA | **86.94 ± 7.19^b^** | **82.71 ± 77.32^b^** | **77.32 ± 6.04^b^** | 54.58 ± 15.744^a^ | **38.46 ± 7.86^c^** | **29.70 ± 17.10^c^** | 41.15 ± 12.87^ac^ |
| Aconitate | 2754.10 ± 713.83^a^ | 3210.59 ± 276.55^a^ | 3493.71 ± 393.75^a^ | 3613.72 ± 902.13^ac^ | **4453.99 ± 590.05^c^** | **4573.96 ± 700.63^c^** | **4220.98 ± 915.78^c^** |
| Alanine | **4931.78 ± 129.84^b^** | 5446.78 ± 773.79^ab^ | 6124.59 ± 322.22^a^ | 5757.27 ± 546.31^a^ | **7095.14 ± 453.57^c^** | **7589.52 ± 597.03^c^** | **7186.99 ± 1257.59^c^** |
| AMP | 2178.56 ± 809.27^a^ | 1734.05 ±269.6^a^ | 1954.39 ± 498.04^a^ | 1424.01 ± 408.69^a^ | 1568.99 ± 387.52^a^ | 1980.35 ± 387.52^a^ | 1851.10 ± 341.53^a^ |
| Arginine | 1358.01 ± 367.92^a^ | 1440.47 ± 307.67^a^ | 1594.13 ± 450.36^a^ | 1698.54 ± 419.34^a^ | 1478.54 ± 262.34^a^ | 1288.99 ± 383.93^a^ | 1160.71 ± 267.49^a^ |
| Asparagine | 4143.71 ± 430.32^a^ | 4816.02 ± 927.75^a^ | 4922.18 ± 457.53^a^ | 5491.46 ± 979.22^a^ | 4508.90± 430.88^a^ | 4062.29 ± 1168.40^a^ | 4000.45 ± 779.43^a^ |
| Citrate | **9710.44 ± 1966.83^b^** | **10140.88 ± 881.71^b^** | **11050.46 ± 869.62^b^** | 13179.15 ± 566.06^a^ | **16943.86 ± 1741.22^c^** | **17274.68 ± 2182.32^c^** | **17782.02 ± 2924.88^c^** |
| Citrulline | 56894.57 ± 13109.11^a^ | 47330.12 ± 5142.14^a^ | 46530.39 ± 8804.66^a^ | 41437.25 ± 5318.67^a^ | 45094.53 ± 3425.93^a^ | 45283.32 ± 9490.64^a^ | 42009.70 ± 3305.33^a^ |
| Cysteine | 5808.76 ± 1210.64^a^ | 6169.45 ± 702.78^a^ | 7444.71 ± 1812.34^a^ | 6707.67 ± 1185.68^a^ | 7138.04 ± 1098.89^a^ | 6389.58 ± 598.47^a^ | 6427.24 ± 778.43^a^ |
| Cystine | **23.25 ±5.65^b^** | **20.46 ± 5.41^b^** | **18.76 ± 4.44^b^** | 15.75 ± 3.20^a^ | 15.28 ± 3.58^a^ | **11.27 ± 2.46^c^** | **10.84 ± 1.88^c^** |
| Fumarate | **1507.69 ± 310.44^b^** | 2140.43 ±395.32^ab^ | **1780.82 ± 461.14^b^** | 2429.35 ± 379.06^a^ | **1897.18 ± 313.35^b^** | **1920.82 ± 301.28^b^** | **1682.45 ± 476.31^b^** |
| GABA | 225.32 ± 97.69^a^ | 274.87 ± 87.89^a^ | 377.56 ± 96.13^a^ | 358.52 ± 128.29^a^ | 347.33 ± 214.81^a^ | 337.59 ± 200.61^a^ | 260.92 ± 97.29^a^ |
| Glutamate | 56894.59 ± 13109.11^a^ | 47330.12 ± 5142.14^a^ | 46530.39 ± 8804.66^a^ | 41437.25 ± 5318.67^a^ | 45094.53 ± 3425.92^a^ | 45283.32 ± 9490.64^a^ | 42009.70 ± 3305.33^a^ |
| Glutamine | 44557.82 ± 6912.19^a^ | 40982.37 ± 3583.99^a^ | 45748.10 ± 4260.41^a^ | 49502.05 ± 8322.49^a^ | 46542.89 ± 1939.36^a^ | 45381.72 ± 7699.53^a^ | 38783.80± 5182.57^a^ |
| Glycine | 1475.93 ± 252.54^a^ | 1365.98 ± 193.24^a^ | 1338.77 ± 129.42^a^ | 1413.01 ± 268.00^a^ | 1408.51 ± 186.99^a^ | 1453.26 ± 305.43^a^ | 1387.74 ± 329.99^a^ |
| Histidine | 265.11 ± 17.12^a^ | 312.46 ± 34.93^a^ | 317.85 ± 21.30^a^ | 355.90 ± 47.10^a^ | 316.26 ± 49.13^a^ | 323.66 ± 71.21^a^ | 314.79 ± 56.50^a^ |
| Isocitrate | **9359.07 ± 1011.51^b^** | **10306.22 ± 951.74^b^** | **11540.69 ± 861.15^b^** | 12761.97 ± 1268.35^a^ | **15920.15 ± 1546.35^c^** | **15968.42 ± 2118.45^c^** | **16666.64 ± 2192.15^c^** |
| Isoleucine | 319.84 ± 61.51^a^ | 362.26 ± 69.26^a^ | 368.26± 82.57^a^ | 384.66 ± 59.91^a^ | 325.96 ± 47.74^a^ | 346.57 ± 95.55^a^ | 328.82 ± 67.08^a^ |
| Lactate | **10112.69 ± 1104.84^b^** | **10319.02 ± 744.94^b^** | **11322.86 ± 876.12^b^** | 13725.08 ± 1385.62^a^ | **17645.74 ± 1813.35^c^** | **17990.27 ± 2272.72^c^** | **18518.62 ± 2600.99^c^** |
| L-Arg succinic acid | **22.15 ± 5.83^b^** | **19.53 ± 2.69^b^** | **20.04 ± 1.83^b^** | 29.88 ± 3.94^a^ | **20.49 ± 3.66^b^** | **18.25 ± 3.20^b^** | **16.4 ± 1.65^b^** |
| Leucine | 306.68 ± 59.44^a^ | 384.13 ± 76.44^a^ | 362.84 ± 84.50^a^ | 375.44 ± 82.09^a^ | 306.58 ± 58.77^a^ | 346.92 ± 99.10^a^ | 309.64 ± 76.42^a^ |
| Lysine | **51030.16 ± 8602.32^b^** | 46903.89 ± 4523.16^a^ | 52501.26 ± 4870.72^a^ | 56406.97 ± 9179.43^a^ | 53142.93 ± 2248.53^a^ | 48076.16 ± 13981.99^a^ | 44722.09 ± 5803.52^a^ |
| Malate | **100890.11 ± 4794.20^b^** | **128883.34 ± 12481.41^b^** | **117954.73 ± 6698.90^b^** | 154926.93 ± 5832.78^a^ | 153800.31 ± 5888.59^a^ | **170983.67 ± 4788.51^c^** | **175962.43 ± 11303.01^c^** |
| Methionine | 550.50 ± 116.19^a^ | 448.87 ± 76.58^a^ | 464.89 ± 50.34^a^ | 517.14 ± 100.66^a^ | 576.61 ± 63.52^a^ | 508.67 ± 161.24^a^ | 522.09 ± 96.01^a^ |
| NAD | 246.22 ± 22.06^a^ | 246.24 ± 34.41^a^ | 209.23 ± 50.31^a^ | 287.86 ± 44.85^ab^ | 344.29 ± 60.32^b^ | 346.13 ±50.24^b^ | 299.53 ± 30.22^b^ |
| Ornithine | 174.23 ± 27.28^a^ | 169.76 ± 35.83^a^ | 167.06 ± 17.88^a^ | 185.84 ± 40.86^a^ | 151.88 ± 15.16^a^ | 133.07 ± 39.49^a^ | 127.78 ± 23.47^a^ |
| Phenylalanine | 1920.20 ± 408.50^a^ | 1987.99 ± 383.68^a^ | 2155.27 ± 502.93^a^ | 2011.63 ± 258.12^a^ | 1804.22 ± 306.66^a^ | 2011.00 ± 542.23^a^ | 1863.67 ± 482.59^a^ |
| Proline | 7404.10 ± 4465.44^ab^ | 13730.77± 6990.52^ab^ | 9183.13 ± 5580.81^ab^ | 16081.86 ± 6754.23^a^ | 12872.53 ± 6216.91^ab^ | **6058.79 ± 2383.81^b^** | **6442.07 ± 3062.62^b^** |
| Serine | 10146.54 ± 1601.52^a^ | 9951.51 ± 1691.72^a^ | 9912.75 ± 1276.59^a^ | 10087.77 ± 633.51^a^ | 9541.70 ± 1794.58^a^ | 9673.16 ± 1331.07^a^ | 10443.10 ± 2360.14^a^ |
| Succinate | **200.64 ± 24.14^b^** | **305.26 ± 41.96^c^** | **270.45 ± 70.27^bc^** | 404.65 ± 63.11^a^ | **288.22 ± 38.79^bc^** | **293.16 ± 71.70^bc^** | **263.26 ± 87.43^bc^** |
| Threonine | 6986.76 ± 954.96^a^ | 6550.99 ± 585.58^a^ | 7383.66 ± 1128.46^a^ | 6695.67 ± 1021.55^a^ | 7127.99 ± 379.77^a^ | 7312.22 ± 1138.18^a^ | 7036.77 ± 870.30^a^ |
| Tryptophan | 252.19 ± 42.58^a^ | 305.25 ± 51.50^a^ | 273.32± 23.17^a^ | 307.33 ± 64.98^a^ | 237.88 ± 39.47^a^ | 252.63 ± 79.44^a^ | 254.38 ± 42.45^a^ |
| Tyrosine | **115.82 ± 18.52^b^** | **118.51 ± 16.54^b^** | **119.75 ± 6.72^b^** | 96.84 ± 14.75^a^ | 86.78 ± 22.09^ac^ | **84.11 ± 15.22^c^** | 93.03 ± 12.81^ac^ |
| Valine | 225.32 ± 97.69^a^ | 274.87 ± 87.89^a^ | 377.56 ± 96.13^a^ | 358.52 ± 128.29^a^ | 347.34 ± 214.81^a^ | 337.59 ± 200.61^a^ | 260.92 ± 97.29^a^ |
| Total AAs | **271513.3 ± 28580.4^b^** | 254674.3 ± 17867.1^ab^ | **251666.9 ± 11728.4^b^** | 232065.5 ± 13637.7^a^ | 231977.0 ± 14938.5^abc^ | **212502.2 ± 13603.4^c^** | **205638.8 ± 15105.6^c^** |
| Total OAs | **129751.8 ± 9976.9^c^** | **163534.8 ± 13250.7^b^** | **160207.1 ± 14629.5^b^** | 199029.1 ± 9697.6^a^ | 194965.9 ± 13126.0^a^ | 215609.6 ± 19762.4^a^ | 215178.1 ± 46209.9^a^ |

**Table S4** Summary of the numerical values of the loadings on the first three PCs of the PCA shown in Figure 4 (C and D) are given. Loadings with the absolute values > ± 0.2 (strong impact) are shown in bold.

| **Metabolite** | **PC1** | **PC2** | **PC3** |
| --- | --- | --- | --- |
| Total organic acids | **-0.44632** | 0 | 0 |
| Fructose | **0.36208** | 0 | 0 |
| Glucose | **0.33913** | 0 | 0 |
| Citrate | **-0.32666** | 0 | 0 |
| Lactate | **-0.32666** | 0 | 0 |
| Isocitrate | **-0.31159** | 0 | 0 |
| Sucrose | **0.28589** | 0 | 0 |
| Total amino acids | **0.27658** | 0 | 0 |
| Starch | **0.27106** | 0 | 0 |
| Malate | -0.11172 | 0 | -0.043679 |
| Succinate | 0 | **-0.58325** | 0 |
| Aconitate | 0 | **-0.58325** | 0 |
| Proline | 0 | **-0.33807** | 0 |
| Fumarate | 0 | **-0.30599** | 0 |
| Asparagine | 0 | **-0.22023** | 0 |
| AMP | 0 | 0.17414 | 0 |
| Alanine | 0 | 0.13506 | 0 |
| Histidine | 0 | -0.098297 | 0 |
| Glutamate | 0 | 0.06054 | 0 |
| Citrulline | 0 | 0.03658 | 0 |
| Arginine | 0 | 0 | **0.26612** |
| Cysteine | 0 | 0 | **0.23254** |
| Cystine | 0 | 0 | 0.11401 |
| Glutamine | 0 | 0 | **0.73563** |
| Glycine | 0 | 0 | 0 |
| Isoleucine | 0 | 0 | 0 |
| Leucine | 0 | 0 | 0 |
| Lysine | 0 | 0 | **0.49949** |
| Methionine | 0 | 0 | 0 |
| Phenylalanine | 0 | 0 | 0 |
| Serine | 0 | 0 | -0.12492 |
| Threonine | 0 | 0 | 0 |
| Tryptophan | 0 | 0 | 0 |
| Tyrosine | 0 | 0 | -0.021008 |
| Valine | 0 | 0 | 0.030927 |
| GABA | 0 | 0 | 0 |
| 2-PG | 0 | 0 | 0 |
| 3-PGA | 0 | 0 | 0 |
| L-Argininosuccinic acid | 0 | 0 | **0.22923** |
| NAD | 0 | 0 | 0 |
| Ornithine | 0 | 0 | 0 |

**Supplemental Table S5.** Primers used for PCR amplification of genomic DNA and cDNA.

Underlined sequences indicate the introduced *BamHI*, *SacI* and *PstI* sites in the primers used to produce expression constructs. ATG in bold print highlight the start codon for methionine.

| Stock Number | Name | Sequence (5´-to3´) |
| --- | --- | --- |
| P950 | *At*GC1_S1141_SacI | GAGCTCATGGTTGCAACAGAGAGGATGAAT |
| P951 | *At*GC1-AS-BamHI | GGATCCATTTCTTGAGTAGTGATTTTGAAG |
| P965 | *Fp*GDCH-S-PstI | CTGCAG**ATG**GCTCTTAGAATCTGGGCT |
| P966 | *Fp*GDCH-AS-PstI | CTGCAGCTACGTGAGCAGAATCTTCTTC |
| P807 | 35STer | CTCGAGAGTATCGATCTGGATTTTAGT |
| P444 | *S16*-forward | GGCGACACAACCAGCTACTGA |
| P445 | *S16*-revers | CGGTAACTCTTCTGGTAACGA |
|  |  |  |

**References**

**Boyes DC, Zayed AM, Ascenzi R, McCaskill AJ, Hoffman NE, Davis KR, Görlach J** (2001) Growth stage-based phenotypic analysis of Arabidopsis: a model for high throughput functional genomics in plants. *The Plant Cell* 13: 1499–1510
